# The Safety of Dronabinol and Nabilone: A Systematic Review and Meta-Analysis of Clinical Trials

**DOI:** 10.3390/ph15010100

**Published:** 2022-01-14

**Authors:** Ákos Bajtel, Tivadar Kiss, Barbara Tóth, Szabolcs Kiss, Péter Hegyi, Nóra Vörhendi, Boglárka Csupor-Löffler, Noémi Gede, Judit Hohmann, Dezső Csupor

**Affiliations:** 1 Department of Pharmacognosy, Faculty of Pharmacy, University of Szeged, 6720 Szeged, Hungary; bajtel.akos@pharmacognosy.hu (Á.B.); kiss.tivadar@szte.hu (T.K.); toth.barbara@szte.hu (B.T.); hohmann.judit@szte.hu (J.H.); 2 Institute for Translational Medicine, Szentágothai Research Centre, Medical School, University of Pécs, 7624 Pécs, Hungary; kissszabolcs1995@gmail.com (S.K.); hegyi2009@gmail.com (P.H.); vorinocci@gmail.com (N.V.); csupor.boglarka@pharmacognosy.hu (B.C.-L.); gede.noemi@gmail.com (N.G.); 3 Centre for Translational Medicine, Semmelweis University, 1085 Budapest, Hungary; 4 Division of Pancreatic Diseases, Heart and Vascular Center, Semmelweis University, 1085 Budapest, Hungary; 5 Department of Clinical Pharmacy, Faculty of Pharmacy, University of Szeged, 6720 Szeged, Hungary

**Keywords:** nabilone, dronabinol, cannabinoid, adverse effects, safety, *Cannabis*, meta-analysis

## Abstract

Dronabinol, a natural cannabinoid, and its semi-synthetic derivative, nabilone, are marketed as medicines in several countries. The aim of our work was to systematically evaluate the frequency of adverse events related to dronabinol or nabilone treatment compared to placebo. Scientific databases were searched for placebo-controlled clinical studies of patients receiving either dronabinol or nabilone therapy with placebo control groups. This meta-analysis was reported following the PRISMA guidelines using the PICO format, and it was registered with the PROSPERO register. There were 16 trials included in the meta-analysis. In the nabilone studies, drowsiness was more than 7 times as frequent in patients treated with nabilone than in the placebo group (OR: 7.25; 95% CI: 1.64–31.95), and the risk of dizziness (OR: 21.14; 95% CI: 2.92–152.75) and dry mouth was also higher (OR: 17.23; 95% CI: 4.33–68.55). The frequency of headache was not different in the two groups. In case of dronabinol, the frequency of dry mouth (OR: 5.58; 95% CI: 3.19–9.78), dizziness (OR: 4.60 95% CI: 2.39–8.83) and headache (OR: 2.90; 95% CI: 1.07–7.85) was significantly higher in the dronabinol groups, whereas in case of nausea, drowsiness and fatigue there was no difference. The severity of adverse events was typically mild-to-moderate and transient. In a risk-benefit assessment, these adverse effects are acceptable compared to the achievable benefit. However, considering the diversity of the adverse effects, more studies are needed to provide a more accurate assessment on the side effect profiles of these two compounds.

## 1. Introduction

The use of cannabinoids and *Cannabis sativa* either as medicine or as food is increasing. Food supplements containing cannabidiol (CBD) or “full spectrum” extracts are very popular. These products have been promoted for a wide range of health issues, such as insomnia, anxiety, drug addiction, fatigue, and cancer. To date, more than 110 different cannabinoids have been isolated from *C. sativa* [1], and the toxicological profiles of the majority of these is unknown. The main psychoactive constituent of *Cannabis* is (−)-trans-Δ⁹-tetrahydrocannabinol (THC). This compound was approved first in the USA in 1985 [2] and is available in several countries as medicine under the international non-proprietary name (INN) dronabinol. Its medicinal use is based on other effects than psychoactivity, i.e., increase of appetite and antiemetic effect. The therapeutic indication of dronabinol is anorexia associated with weight loss in patients with AIDS and nausea and vomiting associated with cancer chemotherapy in patients who have failed to respond adequately to conventional antiemetic treatments [3]. A derivative of THC, nabilone, was also approved for the latter indication of dronabinol, i.e., nausea and vomiting associated with cancer chemotherapy [4]. Both compounds are available as medicines in several European countries, however none of them passed a centralized authorization procedure (i.e., are not available in all the member states of the European Union). The mechanism of action of these two compounds are similar. Both are partial agonists of CB1 and CB2 receptors, although nabilone is more potent, with a slower time to peak effect [5]. The safety profiles of dronabinol and nabilone are very diverse, ranging from musculoskeletal to cardiovascular and neuropsychiatric symptoms, but due to the similarities of pharmacodynamic profiles, quite similar [6]. The most commonly reported adverse reactions of nabilone are drowsiness, vertigo, dry mouth, euphoria, concentration difficulties, ataxia and headache [4], whereas in case of dronabinol, dizziness, euphoria, nausea, paranoid reaction, somnolence abnormal thinking, vomiting and abdominal pain was reported most frequently [3]. However, the incidence of the different adverse events (AEs) in the clinical trials has not been assessed independently and the AE profiles of these cannabinoids has not been compared so far. The aim of our work was to prepare a systematic review of the literature in order to analyze the AEs of dronabinol and nabilone based on the meta-analysis of placebo-controlled trials.

## 2. Results

### 2.1. Literature Search and Study Selection

**Figure 1:**
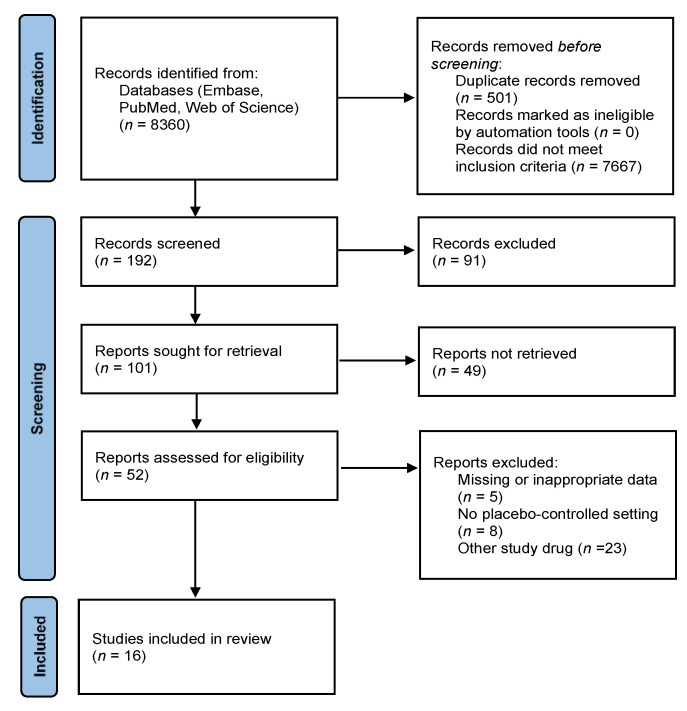
PRISMA flow diagram.

By using the search terms dronabinol and nabilone for the literature search of the EMBASE, PubMed and Web of Science databases and the Cochrane Central Register of Controlled Trials, removing duplicates, the search yielded a total of 7859 potentially relevant reports. The included RCTs were selected according to the flow chart presented in Figure 1. After screening titles, 192 publications remained, and by further screening abstracts, 101 hits were retrieved for full-text screening, of which 85 RCTs were also excluded. The reasons for excluding articles are listed in Supplementary Table S2. Briefly, 26 papers were excluded since these did not report clinical trials, 22 trials were not placebo-controlled, 15 were excluded due to missing or inappropriate data, whereas in 22 trials other study drugs were used than nabilone or dronabinol. A total of 19 RCTs were considered to be appropriate for quantitative analysis [7,8,9,10,11,12,13,14,15,16,17,18,19,20,21,22,23,24,25], and 16 of these were included in the meta-analysis. Although three studies were considered for inclusion, the criterion of the minimal number of studies with the same outcome was not fulfilled in any of the outcomes reported; therefore, these were excluded from the meta-analysis. In 6 studies, nabilone was the study drug (Table 1) [11,13,15,18,22,23], whereas in 10 studies (Table 2) [7,8,9,10,12,16,17,19,21,25], dronabinol was used.

### 2.2. Risk of Bias Assessment

**Figure 2:**
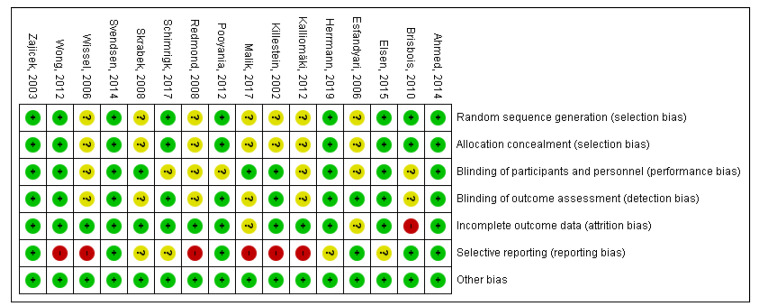
Table of biases.

Overall, the methodical quality of the trials included in our final quantitative analysis was considered to be good, mostly with low or unclear risk of bias (Figure 2). None of the studies showed high risk of selection bias. In nine studies, random sequences or codes were generated by computer programs [10,12,16,17,19,21,22,23,25]. Therefore, these studies were judged to have a low risk of selection bias. However, the remaining seven studies had unclear risk of selection bias [7,8,9,11,13,15,18] because the authors failed to describe the methods used for randomization in detail. Based on the blinding of the personnel and participants and making the interventions as identical as possible, nine studies were considered to have low risk of performance bias [7,8,10,16,17,18,19,21,22]. In the remaining studies [9,11,12,13,15,23,25], it was not mentioned whether the intervention and the comparator were identical in size, shape, color and odor. Moreover, the authors of four of these studies failed to describe precisely who exactly was blinded [11,13,15,25]. Ten trials had low risk of detection bias [7,9,10,12,16,17,19,21,22,23]. In these studies, the assessment of the outcomes was done in a properly blinded manner. However, six trials were judged to have unclear risk of detection bias [8,11,13,15,18,25] because blinding of the outcome assessment were not described in detail, and it was unclear whether the person responsible for the assessment was blinded or not. Almost all of the studies showed low risk of attrition bias. However, in one trial more than half of the enrolled patients did not complete the study [25]; therefore, this study was judged to have a high risk of attrition bias. In the study reported by Esfandyari et al., it is unclear whether there were any patients lost during the course of the trial; hence, the attrition bias of this study is unclear [9]. Furthermore, a relatively high proportion of the enrolled patients did not finish the study of Malik et al., and the underlying reasons were not fully described, so this study also shows an unclear risk of attrition bias [8]. Six studies showed low risk of reporting bias [9,17,19,21,23,25]. In four studies not all the results were clearly indicated numerically [12,16,18,22]; therefore, these studies were judged to have unclear risk of reporting bias. We identified several flaws, for example, inconsistency between the methods and the results section or missing results or *p* values, in six studies; therefore, these studies were considered to have a high risk of reporting bias [7,8,10,11,13,15]. Overall, all the studies showed a low risk of other types of bias. Publication bias was assessed by using Egger’s test, and a funnel plot was utilized for visual assessment. The number of studies allowed this test only in case of headache in dronabinol studies. The inspection of the funnel plot and the significance of Egger’s test (*p* = 0.015) revealed a small study effect in case of this AE (Figure S1).

### 2.3. Study Characteristics

#### 2.3.1. Nabilone

In case of nabilone, 5 of the 6 included trials used a crossover design [11,13,15,22,23]. Clinical trials were performed in Canada (*n* = 4) [11,18,22,23], the UK (*n* = 1) [15] and Austria/Germany/Switzerland (*n* = 1) [13]. Nabilone was used to alleviate agitation in patients with moderate-to-severe Alzheimer’s disease [22], spasticity in people with spinal cord injury [23], spasticity-related pain [13] and fibromyalgia [18]. In two trials the effects of nabilone on capsaicin-induced pain and hyperalgesia [15] were studied, and the analgesic and antihyperalgesic properties on experimental heat pain were studied as well [11]. The duration of these studies was 1–9 weeks. Patients were 18–70 years old (mean age 22.5–50.1 years), except in one trial where patients with Alzheimer’s disease were included and the mean age of the patients was 87 years [22]. The applied dose ranged between 0.5–3 mg daily and in three trials 0.5–1 mg titrating doses were used [15,22,23]. Altogether 154 patients were enrolled and 129 completed the studies.

#### 2.3.2. Dronabinol

Dronabinol was studied in 10 randomized, placebo-controlled trials, performed in Canada (*n* = 1) [25], in Denmark (*n* = 1) [19], in Germany (*n* = 1) [12], in the Netherlands (*n* = 3) [7,16,21], in the USA (*n* = 3) [8,9,10] and in the United Kingdom (*n* = 1) [17], and two of these trials were crossover trials [7,8,9,10,11,12,13,14,15,16,17,18,19,20,21]. Study durations ranged from 2 days to 16 weeks. In the case of one study, dronabinol was administered 4 times, with 2-week washout periods [9]. The enrolled 911 patients were 18–70 years old (mean/median age 26.0–72.1) and in some studies only the mean age was disclosed (46–79 years) [7,16]. The data of 774 patients were assessed. The daily dronabinol dose ranged between 2.5–15 mg. In two trials, the efficacy of dronabinol in the alleviation of neuropathic pain in patients with multiple sclerosis was studied [12,19], and a further trial focused on the efficacy and safety of the drug in multiple sclerosis (MS) patients [7]. In one trial, the effect on gastrointestinal transit and postprandial satiation was studied in healthy human subjects [9], whereas in another trial, the effect on gut transit was studied in patients with irritable bowel syndrome [10]. Malik et al. studied the efficacy in functional chest pain [8], van den Elsen assessed the clinical effect of dronabinol on dementia-related neuropsychiatric symptoms [16], whereas the safety and tolerability of dronabinol was evaluated in elderly people [21]. One study aimed to determine if THC can improve taste and smell perception, appetite, caloric intake and quality of life in cancer patients [25].

### 2.4. Outcomes

#### 2.4.1. Quantitative Analysis—Nabilone

**Figure 3:**
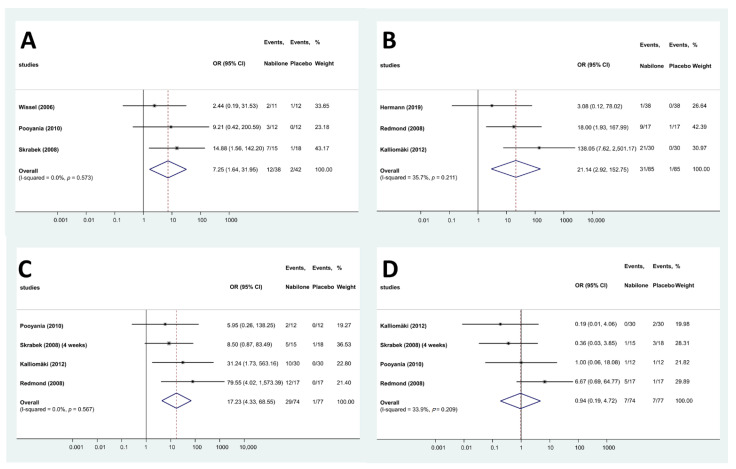
Forest plots of different AEs—nabilone ((**A**): drowsiness; (**B**): dizziness; (**C**): dry mouth; (**D**): headache).

In the studies evaluating the effects of nabilone, 39 different adverse effect were reported (Table S3). These adverse effects were categorized according to the International Statistical Classification of Diseases and Related Health Problems (ICD-10) and split into three main categories [26]: AEs related to the central nervous system, cardiovascular system and miscellaneous. A total of 15 AEs were related to the central nervous system, whereas 5 affected the cardiovascular system. AEs were more frequent in the treated group than in the placebo group in both major types (68 vs. 24 and 25 vs. 6, respectively), and the same applies to the total number of AEs (228 vs. 61). Only 4 AEs (drowsiness, dizziness, headache and dry mouth) were reported in at least three studies and could be meta-analyzed. Drowsiness was more than 7 times frequent in the patients treated with nabilone than in the placebo group (OR: 7.25; 95% CI: 1.64–31.95, Figure 3A), whereas risk of dizziness (OR: 21.14; 95% CI: 2.92–152.75, Figure 3B) and dry mouth was also higher (OR: 17.23; 95% CI: 4.33–68.55, Figure 3C) in the nabilone group. However, the frequency of headache was not different in the two groups (OR: 0.94; 95% CI: 0.19–4.72, Figure 3D). To evaluate the robustness of the results, we performed a leave-one-out sensitivity analysis for each AE by iteratively removing one study at a time and recalculating the summary OR. The summary ORs remained stable in case of the dry mouth and headache, indicating that our results were not driven by any single study, i.e., similar results could be obtained after excluding one study. However, in case of dizziness and drowsiness, no significant difference can be observed for the frequency AEs when leaving out the results of Redmond et al. [11] or Skrabek et al. [18], respectively (Figure S2).

#### 2.4.2. Quantitative Analysis—Dronabinol

**Figure 4:**
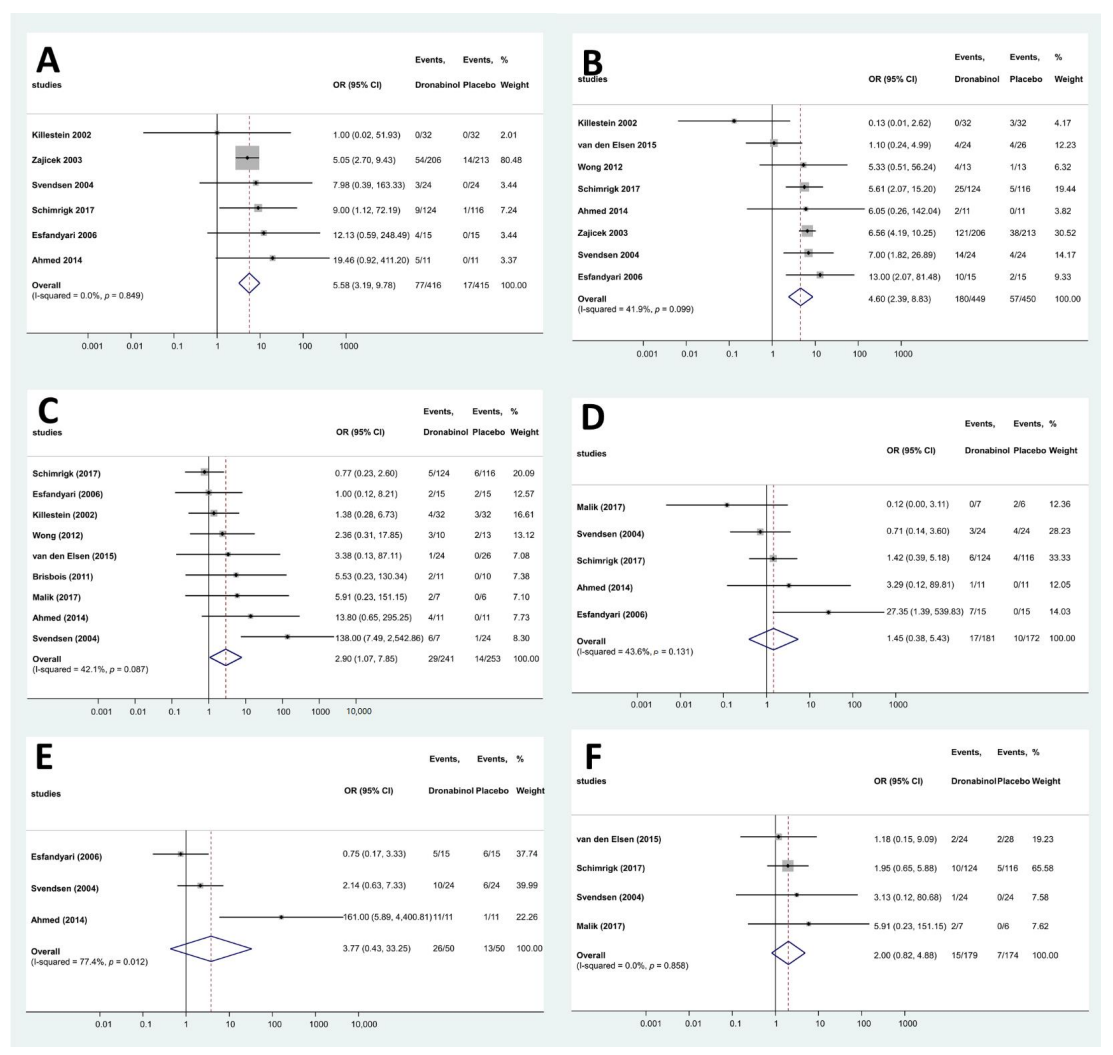
Forest plots of different AEs—dronabinol. ((**A**): dry mouth; (**B**): dizziness; (**C**): headache; (**D**): nausea; (**E**): drowsiness; (**F**): fatigue).

In the analyzed clinical trials, 97 different AEs were reported (Table S4). These were categorized according to the ICD-10 and grouped as AEs affecting the central nervous system, the respiratory system, the musculoskeletal system, the gastrointestinal system, the urogenital system and miscellaneous. The frequency of AEs was higher in these domains in the dronabinol-treated groups (46 vs. 11, 5 vs. 2 and 17 vs. 6, respectively) except for AEs related to the gastrointestinal and urogenital systems. The overall risk of adverse events was higher based on the total number of recorded events (325 vs. 142). Altogether, 6 individual AEs (nausea, drowsiness, dizziness, headache, fatigue and dry mouth) fulfilled the criterium for the meta-analysis. The frequency of dry mouth (OR: 5.58; 95% CI: 3.19–9.78, Figure 4A), dizziness (OR: 4.60 95% CI: 2.39–8.83, Figure 4B) and headache (OR: 2.90; 95% CI: 1.07–7.85, Figure 4C) was significantly higher in the dronabinol groups, whereas in case of nausea, drowsiness and fatigue there was no such difference: (OR: 1.45; 95% CI: 0.38–5.43, Figure 4D), (OR: 3.77; 95% CI: 0.43–33.25, Figure 4E) and (OR: 2.00; 95% CI: 0.82–4.88, Figure 4F), respectively. In addition, sensitivity analyses by iteratively removing one study at a time showed similar and consistent results, thus indicating the robustness of our findings, except for headache, where in case of the removal of the results of either Brisbois et al. [25] or Svendsen et al. [19] or Malik et al. [8] or Ahmed et al. [21], the risk of AEs in groups treated with dronabinol or placebo was not significantly different (Figure S3).

### 2.5. Qualitative Analysis of Excluded Studies

Although three randomized controlled studies were excluded from the meta-analysis, the results of these may also contribute to the whole picture of the AE profile of nabilone and dronabinol. One trial was left out since the number of studies reporting the specific AEs was not sufficient to prepare a meta-analysis [14], whereas one clinical trial was excluded due to inadequate reporting of AEs (using general terms instead of specifying the AEs) [20], and, in one study, the numbers of different AEs were merged and could not be assessed separately [24]. The study of Beaulieu reported the use of nabilone (1 and 2 mg) in patients with postoperative pain compared to placebo and ketoprofen (*n* = 41). The incidence of nausea and vomiting, quality of sleep, euphoria, sedation, pruritus and mood was not different between the study groups. Sedation scores were higher in the 2 mg nabilone group compared to the ketoprofen group, and although euphoria was not significantly different between the four groups, it was more frequent in the nabilone groups [14]. In case of dronabinol, two studies were left out. Van den Elsen et al. assessed efficacy and safety of 1.5 or 3 mg dronabinol compared to placebo in patients with dementia suffering from neuropsychiatric symptoms in a crossover trial. A total of 184 mild to moderate AEs were recorded which were similarly distributed in the THC (91 AEs) and placebo (93 AEs) groups. There was no increase in occurrence of AEs after administering higher doses of dronabinol [20]. Zajicek et al. conducted a study with patients suffering from primary or secondary progressive multiple sclerosis (*n* = 498). Patients received dronabinol (titrated against body weight and AEs, maximum dose 28 mg daily) or placebo for 36 months. Of the patients who received dronabinol 35% had at least one serious AE, compared with 28% of the patients who received placebo. The number and nature of serious AEs did not significantly differ between these 2 groups [24].

## 3. Discussion

The number and importance of cannabinoid-based medicines is increasing in evidence-based medicine. The efficacy of nabilone and dronabinol has been confirmed in several clinical trials and meta-analyses [27,28]. However, data on safety and AEs are also necessary for the assessment of risk benefit ratios. Here, we present the results of the first systematic review and meta-analysis on the AE profiles of nabilone and dronabinol based on the results of randomized, double blind, placebo-controlled trials. In case of nabilone, four AEs were meta-analyzed. Drowsiness, dizziness, and dry mouth were more frequent in the patients treated with nabilone than in the placebo group, whereas the frequency of headache was not different in the two groups. In patients treated with dronabinol, more adverse effects could be meta-analyzed. The frequency of dizziness, dry mouth and headache was significantly higher in the dronabinol groups, whereas in case of nausea, drowsiness and fatigue no significant difference could be observed. Dizziness and dry mouth are common in case of the application of both pharmaceuticals. This might be surprising based on the similar mechanism of action of the two compounds, however, the measures of agonist activities on CB1 and CB2 receptors are different, and both compounds might also act on other targets that also affect adverse effect profiles. The adverse effects discussed here are diverse, but not severe. In the analyzed clinical trials, 40 different adverse effects were reported for nabilone and 111 for dronabinol; however, the majority of these were not recorded in at least 3 trials that would be sufficient for meta-analysis. In the case of radiotherapy-induced nausea and vomiting, international guidelines recommend the use of serotonin receptor antagonists (e.g., granisetron, ondansetron, tropisetron) and dexamethasone as prophylaxis [29]. In case of chemotherapy-induced nausea and vomiting, the recommendations are more diverse; however, serotonin receptor antagonists and dexamethasone are the most commonly used medications [30]. The long-term use of dexamethasone is related to several adverse events, whereas in the case of serotonin receptor antagonists, the most frequent adverse effects are headache, constipation, weakness and somnolence [31]. Although the side effect profiles of cannabinoids have not been clinically compared with the therapies recommended by guidelines, based on the available evidence, the benefit–risk ratio of cannabinoids does not seem to be inferior. In the case of anorexia associated with weight loss in patients with AIDS, the lack of other pharmaceuticals with confirmed clinical efficacy makes dronabinol an indispensable part of the therapy, and this fact has to be taken into account in the benefit–risk assessment. Further, in high-quality trials of appropriate patient size, examining the side-effects of dronabinol or nabilone with comparable and more uniform endpoints would allow an assessment of the safety profile of these compounds with a lower risk of bias. Moreover, a considerable amount of trials reporting the same or similar side-effects that can be easily grouped and that are related to different doses of these drugs would enable the assessment of the dose dependency of the side-effects.

## 4. Materials and Methods

The following PICO (patients, intervention, comparison, outcome) format was applied: P: adult patients; I: dronabinol or nabilone; C: placebo; and O: frequency of adverse effects. The meta-analysis was reported according to the PRISMA statement. The meta-analysis protocol was registered in the International Prospective Register of Systematic Reviews (PROSPERO) a priori (registration number CRD42021240190).

### 4.1. Search Strategy

Literature search was conducted until 21 February 2020, by using the following search strategy: [dronabinol OR nabilone] for EMBASE; [(“dronabinol”[MeSH Terms] [All Fields]) OR (“nabilone”[MeSH Terms] [All Fields])] for PubMed; [dronabinol OR nabilone] for Cochrane Central Register of Controlled Trials; and [TOPIC: (dronabinol OR nabilone) Timespan: All years. Indices: SCI-EXPANDED, SSCI, A&HCI, ESCI] for Web of Science. No publication date or publication status or language restrictions were applied. For transparency, the meta-analysis was based on publicly available data, neither the authors of articles nor the manufacturers of studied products were contacted for additional information.

### 4.2. Eligibility Criteria

All randomized, placebo-controlled trials (RCTs) evaluating the clinical effects of dronabinol or nabilone and reporting AEs were included. For each outcome, at least 3 clinical trials involving different patient populations were required to perform a statistical analysis.

### 4.3. Study Selection

Record management was performed using the Mendeley 1.17.9 software. After removing duplicates and records without an abstract, the remaining records were screened for eligibility on the basis of article titles and abstracts. The eligibility of the full texts of the remaining records was assessed by two reviewers (AB, TK), independently. Disagreement between reviewers was resolved by discussion or, if necessary, by consulting with a third reviewer (DC).

### 4.4. Data Extraction and Synthesis of the Results

Data collection was executed following the PRISMA guidelines (Table S1). Study characteristics and results were extracted by two reviewers, independently. Discrepancies in extracted data were resolved by discussion. The following data items were extracted from the included papers: study design, sample size and characteristics of the patient population, duration, intervention details and numbers of different AEs.

### 4.5. Risk of Bias

The risk of bias was assessed using the Cochrane Collaboration tool, which includes seven specific domains: random sequence generation, allocation concealment, blinding of participants and personnel, blinding of outcome assessment, incomplete outcome data, selective reporting and other scores of bias. For each domain, studies were judged to have either high (red), unclear (yellow) or low (green) risk of bias. Disagreements in quality of studies were resolved by discussion. A risk of bias summary table and figure were generated by the RevMan 5 software [32].

### 4.6. Statistical Analysis

Pooled odds ratios (ORs) were calculated for dichotomous outcomes. A random-effect model was applied in all analyses with the DerSimonian–Laird estimation. Statistical heterogeneity was analyzed using the I^2^ and χ^2^ tests to gain probability values; *p* < 0.10 was defined to indicate significant heterogeneity. The I^2^ test represents the percentage of total variability across studies because of heterogeneity. I^2^ values of 30–60%, 50–90% and 75–100% corresponded to moderate, substantial and considerable heterogeneity, respectively, based on Cochrane’s handbook [32]. Forest plots displayed the results of the meta-analysis. Sensitivity analyses were also carried out omitting one study and calculating the summary OR, weighted mean difference with the 95% CI to investigate the influence of a single study on the final estimation. Publication bias was assessed by performing Egger’s test, and a funnel plot was utilized for visual assessment [33]. A leave-one-out sensitivity analysis was performed by iteratively removing one study at a time to confirm that our findings were not driven by any single study. The statistical analyses were performed with Stata 16 SE (Stata Corp).

## Figures and Tables

**Table 1:** Summary of nabilone studies.

First Author, Year	Country	Study Drug	Posology	Duration	Enrolled Patients	Patients Who Have Completed the Trial	Mean Age [yrs (SD)]	Sex [M/F (N)]	Outcomes	Reported Adverse Events
Hermann, 2019	Canada	nabilone	1–2 mg once a day	14 weeks	39	33	placebo & active: 87 (10)	30/9	Efficacy and safety of nabilone for agitation with moderate to severe Alzheimer’s	Sedation (including lethargy, treatment limiting sedation, significant increase in NPS, myocardial infarction, bradycardia, rash, dizziness, lethargy
Kalliomäki 2012	UK	nabilone	1–3 mg	7 weeks & 5 days	30	24	placebo & active: 29.3 (no data)	30/0	Effect of nabilone on capsaicin-induced pain and hyperalgesia and on other CNS biomarkers	Somnolence, Postural dizziness, Tachycardia, Bradycardia, Dizziness, Headache, Fatigue, Dry mouth
Pooyania, 2010	Canada	nabilone	0.5 mg once or bid	10 weeks	12	11	placebo & active: 42.36 (no data)	11/0	Alleviation of spasticity in patients with spinal cord injury (SCI)	Ataxia, Drowsiness, Vertigo (mild), Lack of motivation, Headache, Asthenia, Dry mouth
Redmond, 2008	Canada	nabilone	0.5–1 mg	3 visits with washout periods of at least one week	20	17	placebo & active: male: 22.5 (1.5) female: 23.2 (2.8)	7/10	Analgesic and antihyperalgesic properties of nabilone	Mild sedation, Euphoria, Feeling cold, Nausea, Dizziness, Headache, Increased appetite, Dry mouth
Skrabek, 2008	Canada	nabilone	0.5–1 mg bid	4 weeks	40	33	placebo: 50.11 (5.96) active: 47.6 (9.13)	37/3	Benefit of nabilone in pain management and QoL improvement in patients with fibromyalgia	Euphoria, Depression, Psychological high, Dissociation, Nightmares, Decreased concentration, Ataxia, Confusion, Hallucination, Orthostatic hypotension, Tachycardia, Sensory disturbance, Drowsiness, Lightheaded, Vertigo, Headache, Dysphoria, Anorexia, Dry mouth
Wissel, 2006	Austria/Germany	nabilone	0.5 mg once or tid	9 weeks	13	11	placebo & active: 44.85 (13.82)	4/9	Efficacy and safety of low dose nabilone in spasticity related pain	Dysphagia (slight), Drowsiness, Weakness in lower limbs (slight)

F: female, M: male, ND: no data, yrs: years, bid: twice a day, tid: three times daily.

**Table 2:** Summary of dronabinol studies.

First Author, Year	Country	Study Drug	Posology	Duration	Enrolled Patients	Patients Who Have Completed the Trial	Mean Age [yrs (SD)]	Sex [M/F (N)]	Outcomes	Reported Adverse Events
Malik, 2007	USA	dronabinol	5 mg bid for 4 weeks	4 weeks	19	13	placebo: 42 (ND) active: 44 (ND)	2/11	Effect of dronabinol on pain threshold, frequency, and intensity in functional chest pain (FCP)	Loose stools, nausea, headache, fatigue
Schimrigk, 2017	Germany	dronabinol	titration to daily doses 7.5–15.0 mg	16 weeks	240	169	placebo: 47 (9.7) active: 48.4 (9.6)	65/175	Positive benefit–risk ratio of dronabinol in the treatment of neuropathic pain in MS patients	Insomnia, Nausea, Dizziness, Vertigo, Headache, Fatigue, Dry mouth
van den Elsen, 2015	The Netherlands	dronabinol	1.5 mg tid for 3 weeks	3 weeks	50	50	placebo: 78 (7) active: 79 (8)	25/25	Efficacy and safety of THC in the treatment of dementia-related neuropsychiatric symptoms (NPS)	Delirium, Cognitive disorder, Euphoric mood, Bradykinesia, Somnolence, Agitation, Nasopharyngitis, Pneumonia, COPD, Back pain, Muscle weakness, Muscle spasms, Pain in extremity, Renal impairment, Urge incontinence, Dry eye, Eye hemorrhage, Miosis, Balance disorder, Chest pain, Skin disorder, not otherwise specified, Dizziness, Sensory loss, Restlessness, Aphasia, Apraxia, Headache, Fatigue, Malaise, Presyncope, Syncope, Decreased appetite, Increased gamma-glutamyl transferase,
Ahmed, 2014	The Netherlands	dronabinol	3–6.5 mg	6 weeks	12	11	placebo & active: 72.1 (5)	6/6	Safety and tolerability effects of THC in elderly	Euphoria, Concentration problem, Visual hallucination, Relaxation, Dry eye, Blurred vision, Nausea, Coordination disturbance, Drowsiness, Dizziness, Headache, Malaise, Dry mouth
Wong, 2012	USA	dronabinol	2.5 or 5 mg bid	2 days	36	36	placebo: 36.7 (3.1) active (2.5 mg): 47.7 (7.9) active (5 mg): 42.3 (4.5)	2/34	Gut transit in IBS-D and dronabinol’ transit effect	“Loopy”, foggy thinking, Hot flushes, Drowsiness/Tiredness, Dizziness/Light-headedness, Headache
Brisbois, 2011	Canada	dronabinol	2.5 mg bid (patients had the option to increase their drug dose to a maximum of 20 mg/day)	3 weeks & 1 day	46	21	placebo: 65.5 (8) active: 67 (10.9)	12/9	Effects of THC on chemosensory perception	Confusion, Seizure, Troubles sleeping, Pneumonia, Thrush, Stomach cramps, Bowel obstruction/constipation, Diarrhea, Vaginal discharge, Unsteady feet, Shortness of breath/fluid on lungs, Nausea/Vomiting, Hives/Rash, Fever, Headache, Pain, Tired/Drowsy, Oedema, Low blood count
Esfandyari, 2006	USA	dronabinol	5–7.5 mg bid	2 days	30	30 (27) (3 patients did not complete the study; however, their missing data is included in the ITT analysis)	placebo: 29 (1) active: 26 (2)	14/16	Effect of dronabinol of gastrointestinal transit and postprandial satiation	Excitement, Euphoria/Relaxed, Disturbed mental concentration, Nausea, Numbness, Flushing, Drowsiness, Dizziness/Light-headedness, Headache, Vasovagal, Dry mouth
Svendsen, 2004	Denmark	dronabinol	titration to 5 mg bid	3 weeks	24	24	placebo & active: 50 (median)	10/14	Effect of dronabinol on central neuropathic pain in MS patients	Euphoria, Feeling of drunkenness, Speech disorders, Hyperactivity, Nervousness, Aggravated MS, Migraine, Sleep difficulty, Upper airway infection, Muscle weakness, Myalgia, Hot flushes, Diplopia, Balance difficulty, Palpitations, Abdominal pain, Nausea, Drowsiness, Dizziness, Fever, Headache, Fatigue, Anorexia, Weight decrease, Dry mouth, Chills
Zajicek, 2003	UK	dronabinol	2.5 mg	15 weeks	419	404	placebo: 50.9 (7.6) active: 50.2 (8.2)	141/278	Effect of cannabinoids on spasticity and other symptoms in MS patients	Bladder, Depression of anxiety, Dizzy of light-headedness, Dry mouth, Gastrointestinal tract, Improvement in symptoms, Infection, Miscellaneous, Numbness of paresthesia, Pain, Sleep, Spasms of stiffness, Tremor of lack of coordination, Vision, Weakness of reduced mobility
Killestein, 2002	The Netherlands	dronabinol	2.5–5 mg bid	4 weeks	16	16	placebo & active: 46 (7.9)	no data	Efficacy, safety, and tolerability effects of THC in MS patients	Emotional lability, Ataxia, Somnolence, Increased spasticity, Dizziness, Headache, Dry mouth, Other

F: female, M: male, ND: no data, yrs: years, bid: twice a day, tid: three times daily.

## Data Availability

All data generated or analyzed during this study are included in this published article and its supplementary information files.

## Supplementary Materials

The following are available online at https://www.mdpi.com/article/10.3390/ph15010100/s1, Figure S1: Funnel plot of studied recording the frequency of headache in patients treated with dronabinol or placebo, Figure S2: Results of leave-one-out sensitivity analysis for nabilone, Figure S3: Results of leave-one-out sensitivity analysis for dronabinol, Table S1: PRISMA Checklist, Table S2: List of excluded studies with the reason for exclusion, Table S3: Adverse events in the clinical trials with nabilone, Table S4: Adverse events in the clinical trials with dronabinol.

## References

[1] Gülck T., Møller B.L. (2020). Phytocannabinoids: Origins and biosynthesis. Trends Plant Sci..

[2] Todaro B. (2012). Cannabinoids in the treatment of chemotherapy-induced nausea and vomiting. J. Natl. Compr. Cancer Netw..

[3] MARINOL® (Dronabinol). NDA 18-651/S-021. https://www.accessdata.fda.gov/drugsatfda_docs/label/2005/018651s021lbl.pdf.

[4] CESAMET (Nabilone) Capsules. NDA 18-677/S-011. https://www.accessdata.fda.gov/drugsatfda_docs/label/2006/018677s011lbl.pdf.

[5] Wallach J. (2021). Medicinal Cannabis: An overview for health-care providers. Remington.

[6] Fraguas-Sánchez A.I., Torres-Suárez A.I. (2018). Medical use of cannabinoids. Drugs.

[7] Killestein J., Hoogervorst E.L., Reif M., Kalkers N.F., Van Loenen A.C., Staats P.G., Gorter R.W., Uitdehaag B.M., Polman C.H. (2002). Safety, tolerability, and efficacy of orally administered cannabinoids in MS. Neurology.

[8] Malik Z., Bayman L., Valestin J., Rizvi-Toner A., Hashmi S., Schey R. (2016). Dronabinol increases pain threshold in patients with functional chest pain: A pilot double-blind placebo-controlled trial. Dis. Esophagus.

[9] Esfandyari T., Camilleri M., Ferber I., Burton D., Baxter K., Zinsmeister A.R. (2006). Effect of a cannabinoid agonist on gastrointestinal transit and postprandial satiation in healthy human subjects: A randomized, placebo-controlled study. Neurogastroenterol. Motil..

[10] Wong B.S., Camilleri M., Eckert D., Carlson P., Ryks M., Burton D., Zinsmeister A.R. (2012). Randomized pharmacodynamic and pharmacogenetic trial of dronabinol effects on colon transit in irritable bowel syndrome-diarrhea. Neurogastroenterol. Motil..

[11] Redmond W.J., Goffaux P., Potvin S., Marchand S. (2008). Analgesic and antihyperalgesic effects of nabilone on experimental heat pain. Curr. Med Res. Opin..

[12] Schimrigk S., Marziniak M., Neubauer C., Kugler E.M., Werner G., Abramov-Sommariva D. (2017). Dronabinol is a safe long-term treatment option for neuropathic pain patients. Eur. Neurol..

[13] Wissel J., Haydn T., Müller J., Brenneis C., Berger T., Poewe W., Schelosky L.D. (2006). Low dose treatment with the synthetic cannabinoid Nabilone significantly reduces spasticity-related pain: A double-blind placebo-controlled cross-over trial. J. Neurol..

[14] Beaulieu P. (2006). Effects of nabilone, a synthetic cannabinoid, on postoperative pain. Can. J. Anesth..

[15] Kalliomäki J., Philipp A., Baxendale J., Annas P., Karlsten R., Segerdahl M. (2012). Lack of effect of central nervous system-active doses of nabilone on capsaicin-induced pain and hyperalgesia. Clin. Exp. Pharmacol. Physiol..

[16] Elsen G.A.V.D., Ahmed A.I., Verkes R.-J., Kramers C., Feuth T., Rosenberg P.B., Van Der Marck M.A., Rikkert M.G.O. (2015). Tetrahydrocannabinol for neuropsychiatric symptoms in dementia: A randomized controlled trial. Neurology.

[17] Zajicek J., Fox P., Sanders H., Wright D., Vickery J., Nunn A., Thompson A. (2003). Cannabinoids for treatment of spasticity and other symptoms related to multiple sclerosis (CAMS study): Multicentre randomised placebo-controlled trial. Lancet.

[18] Skrabek R.Q., Galimova L., Ethans K., Perry D. (2008). Nabilone for the treatment of pain in fibromyalgia. J. Pain.

[19] Svendsen K.B., Jensen T.S., Bach F.W. (2004). Does the cannabinoid dronabinol reduce central pain in multiple sclerosis? Randomised double blind placebo controlled crossover trial. BMJ.

[20] Elsen G.A.V.D., Ahmed A.I., Verkes R.-J., Feuth T., van der Marck M.A., Rikkert M.G.O. (2015). Tetrahydrocannabinol in behavioral disturbances in dementia: A crossover randomized controlled trial. Am. J. Geriatr. Psychiatry.

[21] Ahmed A.I., Elsen G.A.V.D., Colbers A., van der Marck M.A., Burger D.M., Feuth T.B., Rikkert M.G.O., Kramers C. (2014). Safety and pharmacokinetics of oral delta-9-tetrahydrocannabinol in healthy older subjects: A randomized controlled trial. Eur. Neuropsychopharmacol..

[22] Herrmann N., Ruthirakuhan M., Gallagher D., Verhoeff N.P.L., Kiss A., Black S.E., Lanctôt K.L. (2019). Randomized placebo-controlled trial of nabilone for agitation in Alzheimer’s disease. Am. J. Geriatr. Psychiatry.

[23] Pooyania S., Ethans K., Szturm T., Casey A., Perry D. (2010). A Randomized, double-blinded, crossover pilot study assessing the effect of nabilone on spasticity in persons with spinal cord injury. Arch. Phys. Med. Rehabil..

[24] Zajicek J., Ball S., Wright D., Vickery J., Nunn A., Miller D., Cano M.G., McManus D., Mallik S., Hobart J. (2013). Effect of dronabinol on progression in progressive multiple sclerosis (CUPID): A randomised, placebo-controlled trial. Lancet Neurol..

[25] Brisbois T.D., de Kock I.H., Watanabe S.M., Mirhosseini M., Lamoureux D.C., Chasen M., MacDonald N., Baracos V.E., Wismer W.V. (2011). Delta-9-tetrahydrocannabinol may palliate altered chemosensory perception in cancer patients: Results of a randomized, double-blind, placebo-controlled pilot trial. Ann. Oncol..

[26] World Health Organization (1994). International Statistical Classification of Diseases and Related Health Problems.

[27] Rocha F.C.M., Stéfano S.C., Haiek R.D.C., Oliveira L.M.Q.R., Da Silveira D.X. (2008). Therapeutic use of *Cannabis sativa* on chemotherapy-induced nausea and vomiting among cancer patients: Systematic review and meta-analysis. Eur. J. Cancer Care.

[28] Smith L.A., Azariah F., Lavender V.T., Stoner N.S., Bettiol S. (2015). Cannabinoids for nausea and vomiting in adults with cancer receiving chemotherapy. Cochrane Database Syst. Rev..

[29] McKenzie E., Zaki P., Raman S., Olson R., McFarlane T., DeAngelis C., Chan S., Pidduck W., Razvi Y., Bushehri A. (2019). Radiation-induced nausea and vomiting: A comparison between MASCC/ESMO, ASCO, and NCCN antiemetic guidelines. Support. Care Cancer.

[30] Razvi Y., Chan S., McFarlane T., McKenzie E., Zaki P., DeAngelis C., Pidduck W., Bushehri A., Chow E., Jerzak K.J. (2018). ASCO, NCCN, MASCC/ESMO: A comparison of antiemetic guidelines for the treatment of chemotherapy-induced nausea and vomiting in adult patients. Support. Care Cancer.

[31] Spartinou A., Nyktari V., Papaioannou A. (2017). Granisetron: A review of pharmacokinetics and clinical experience in chemotherapy induced—Nausea and vomiting. Expert Opin. Drug Metab. Toxicol..

[32] Higgins J.P.T., Cochrane Collaboration (2020). Cochrane Handbook for Systematic Reviews of Interventions.

[33] Egger M., Smith G.D., Schneider M., Minder C. (1997). Bias in meta-analysis detected by a simple, graphical test. BMJ.

