# Supplementary material for: The Safety of Dronabinol and Nabilone: A Systematic Review and Meta-Analysis of Clinical Trials
Source: Pharmaceuticals (Basel). 2022 Jan 14;15(1):100. doi: 10.3390/ph15010100 (PMC8778752; doi:10.3390/ph15010100)
Supplement: Supplementary file 1 [file pharmaceuticals-15-00100-s001.zip › pharmaceuticals-1526066-supplementary.pdf]

**Figure S1** Funnel plot of studied recording the frequency of headache in patients treated with dronabinol or placebo

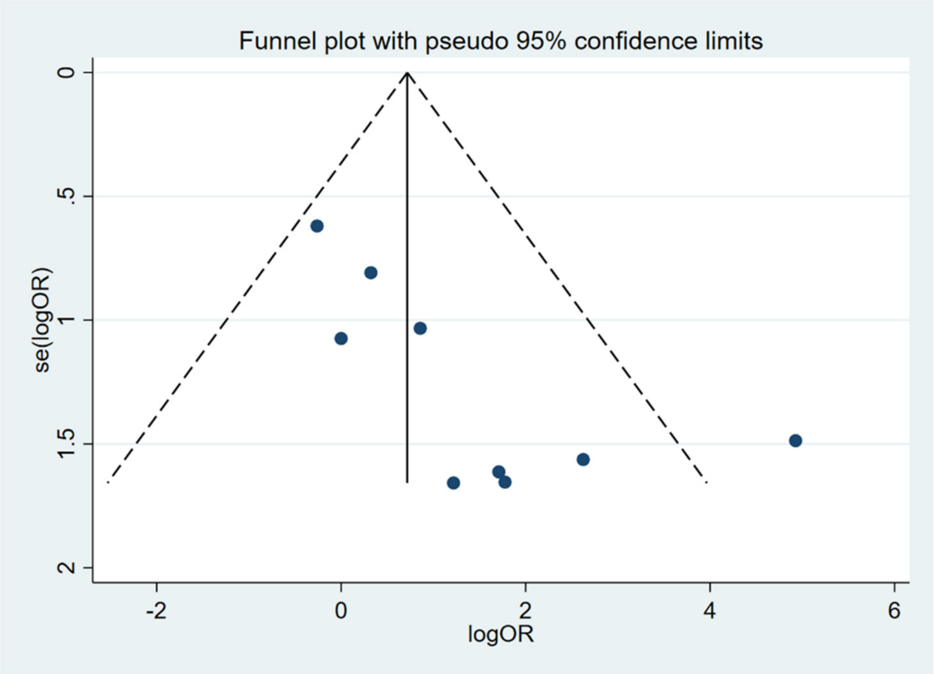

**Figure S2** Results of leave-one-out sensitivity analysis for nabilone. The vertical axis shows the omitted studies, the horizontal axis represents the odds ratio. The circle indicates the pooled OR when the left study is omitted in the meta-analysis, and the broken line represent the respective 95% confidence intervals

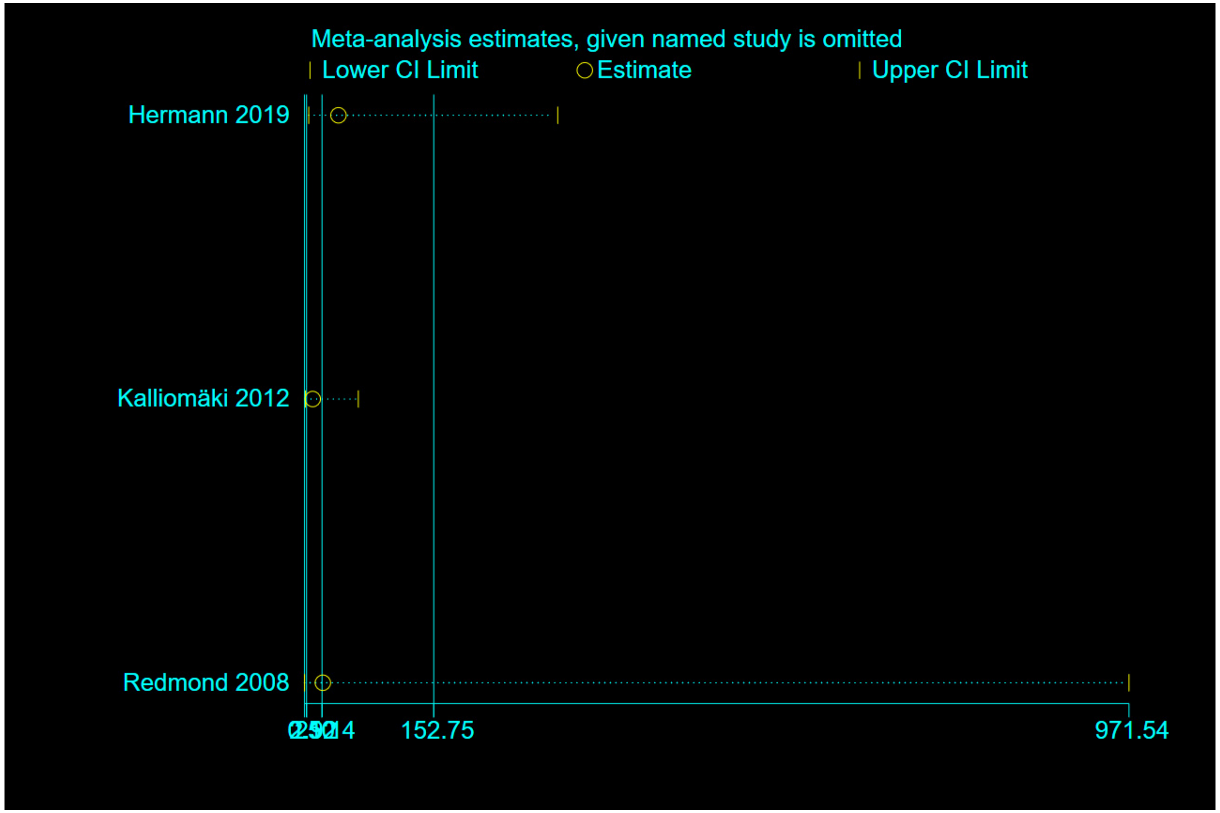


dizziness

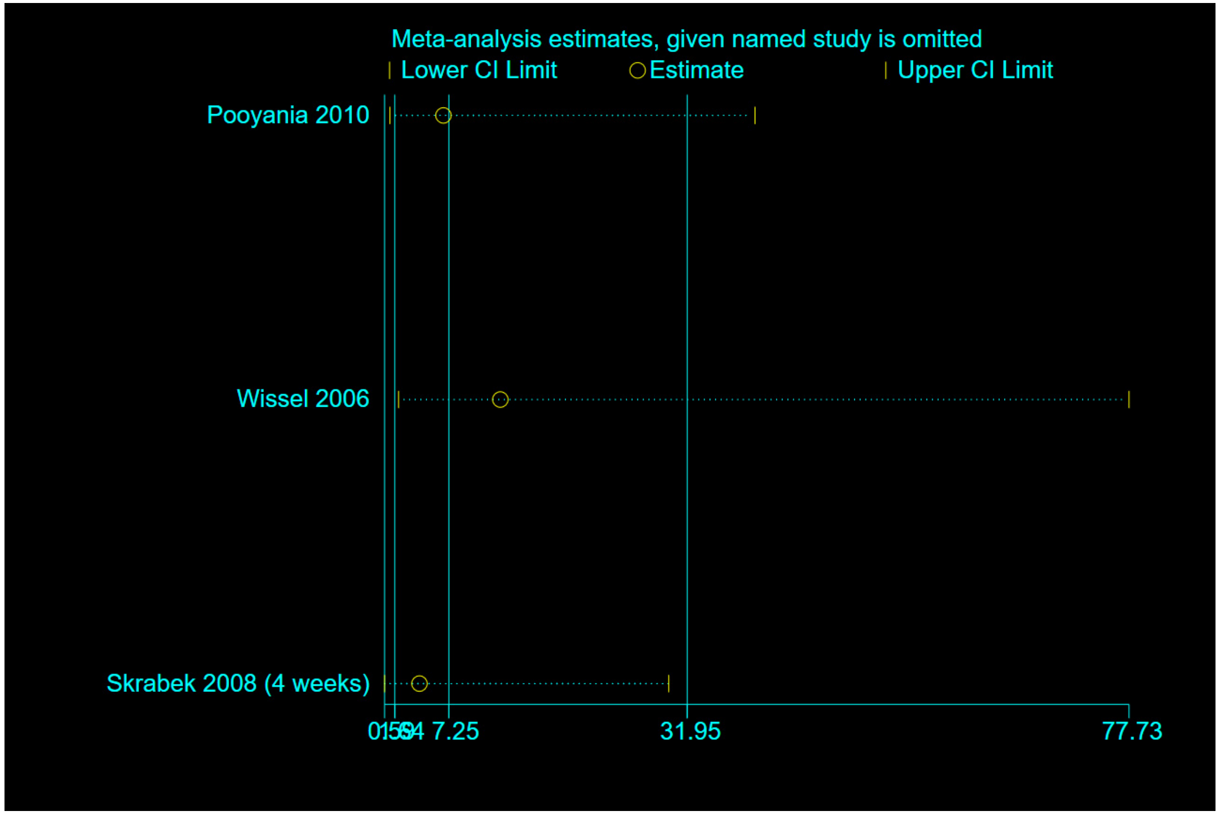
Meta-analysis estimates, given named study is omitted
Lower CI Limit
Estimate
Upper CI Limit
Pooyania 2010
Wissel 2006
Skrabek 2008 (4 weeks)
7.25
31.95
77.73

drowsiness

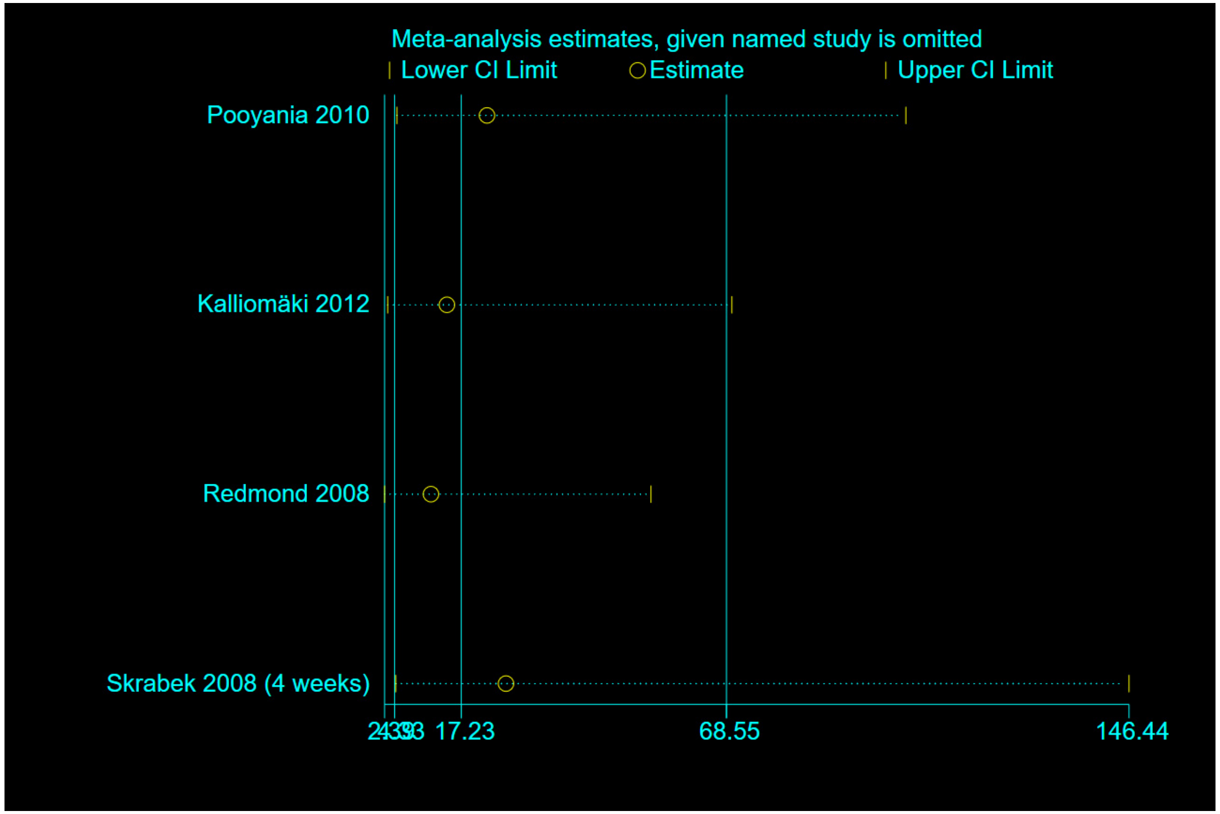
Meta-analysis estimates, given named study is omitted
Lower CI Limit
Estimate
Upper CI Limit
Pooyania 2010
Kalliomäki 2012
Redmond 2008
Skrabek 2008 (4 weeks)
17.23
68.55
146.44

dry mouth

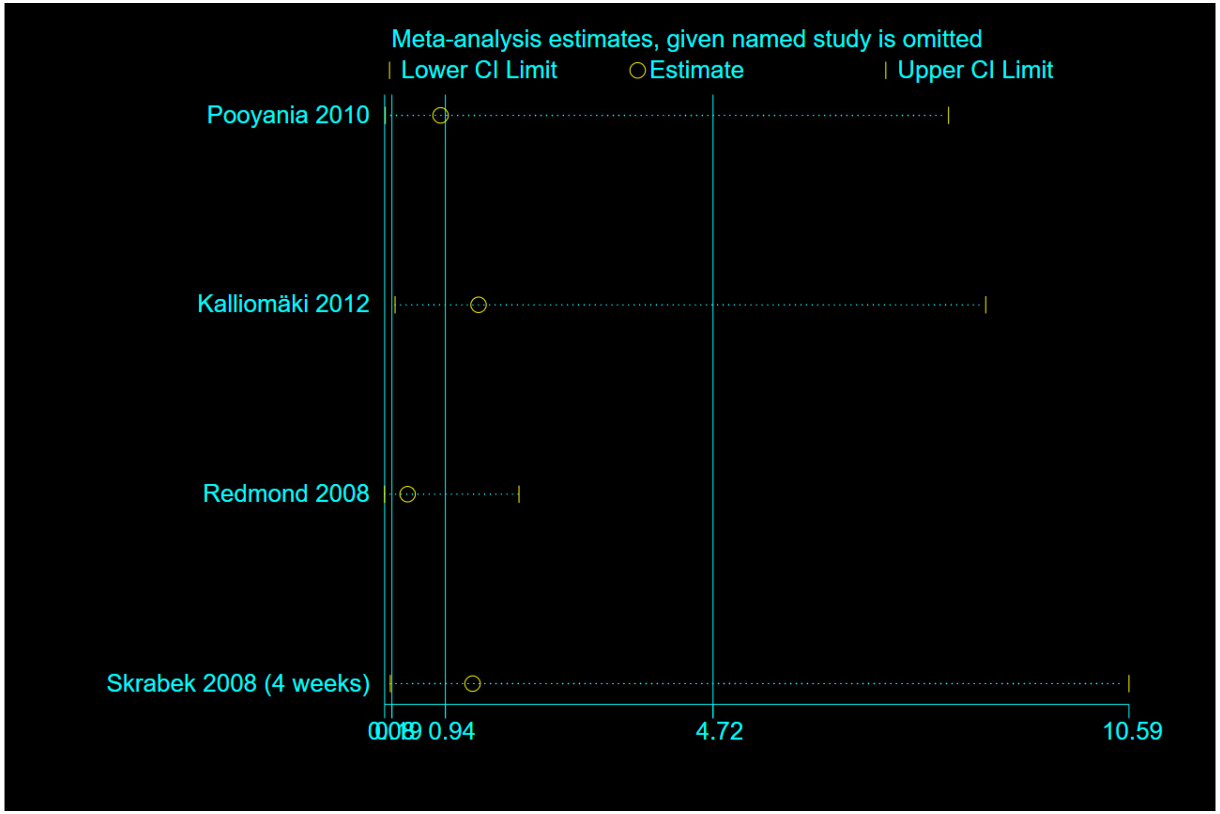
Meta-analysis estimates, given named study is omitted
Lower CI Limit
Estimate
Upper CI Limit
Pooyania 2010
Kalliomäki 2012
Redmond 2008
Skrabek 2008 (4 weeks)
0.94
4.72
10.59

headache

**Figure S3** Results of leave-one-out sensitivity analysis for dronabinol. The vertical axis shows the omitted studies, the horizontal axis represents the odds ratio. The circle indicates the pooled OR when the left study is omitted in the meta-analysis, and the broken line represent the respective 95% confidence intervals

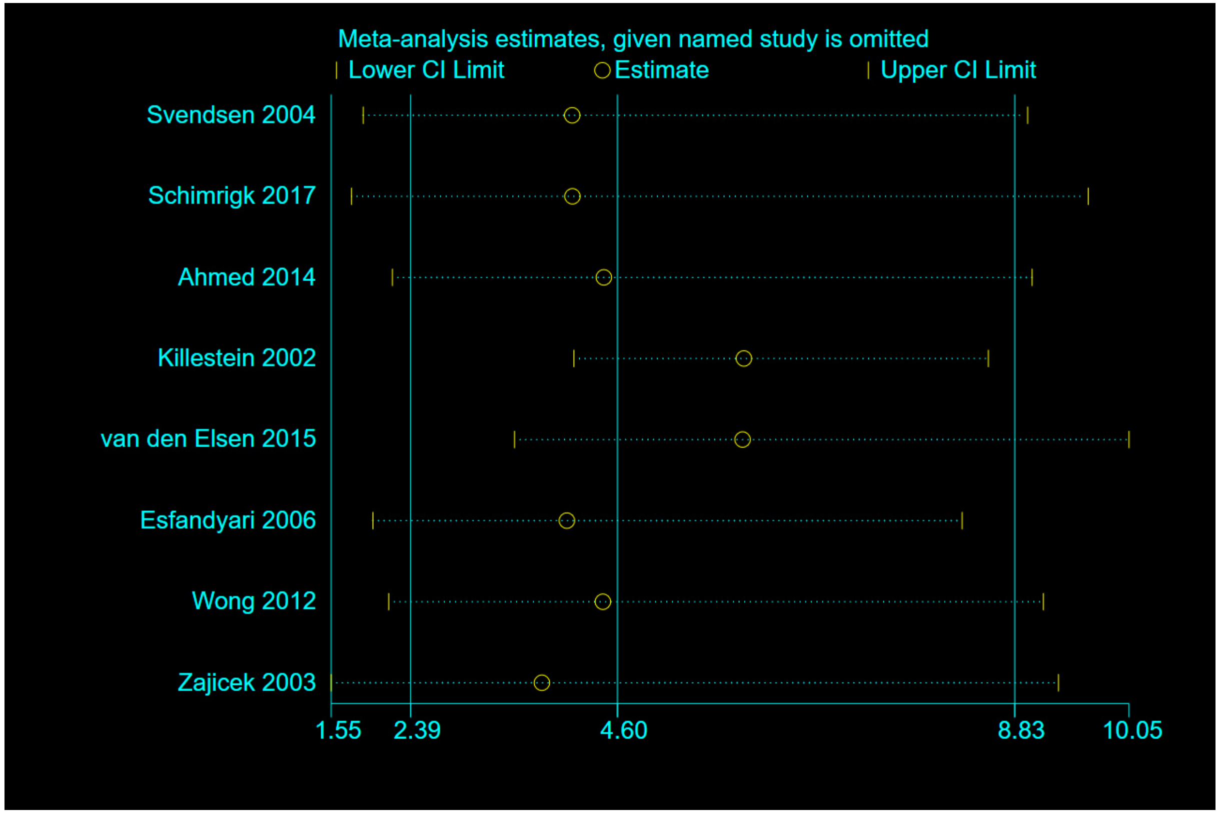


dizziness

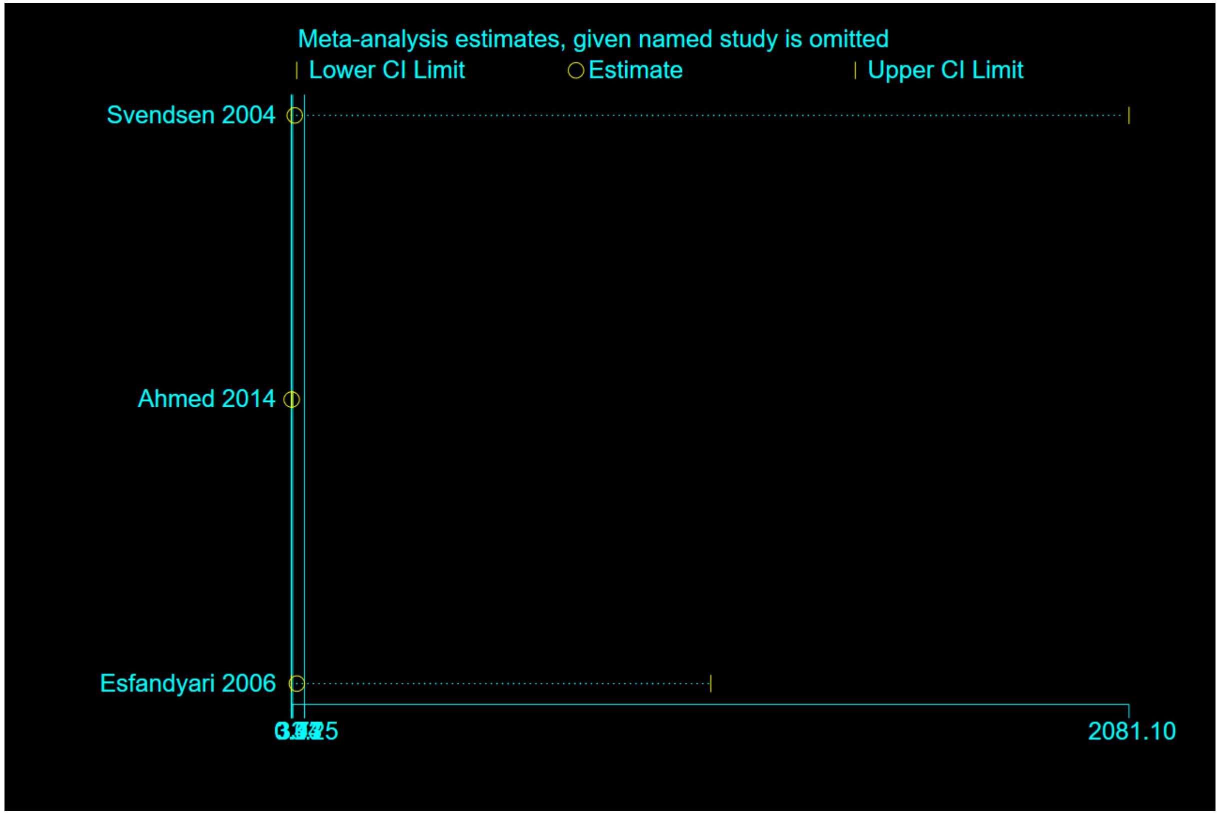
Meta-analysis estimates, given named study is omitted
Lower CI Limit
Estimate
Upper CI Limit
Svendsen 2004
Ahmed 2014
Esfandyari 2006
2081.10

drowsiness

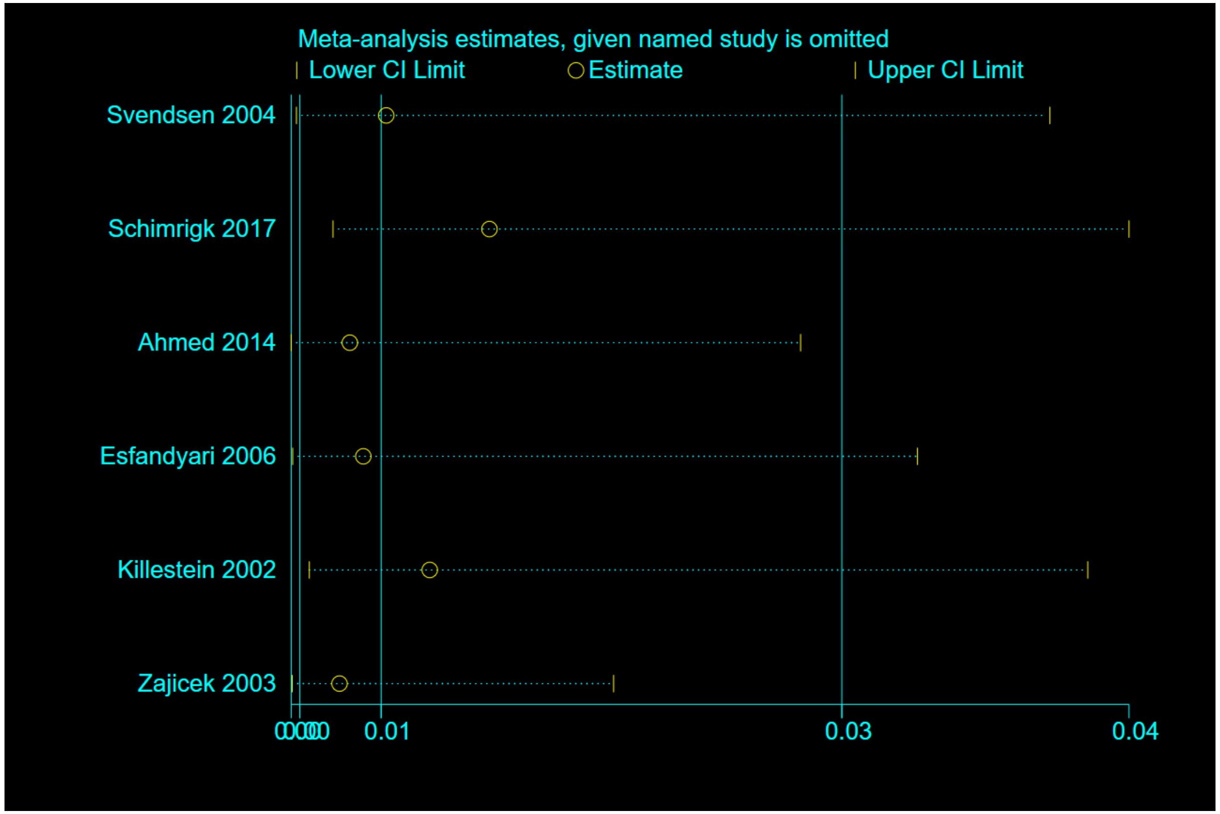
Meta-analysis estimates, given named study is omitted
Lower CI Limit
Estimate
Upper CI Limit
Svendsen 2004
Schimrigk 2017
Ahmed 2014
Esfandyari 2006
Killestein 2002
Zajicek 2003
0.01
0.03
0.04

dry mouth

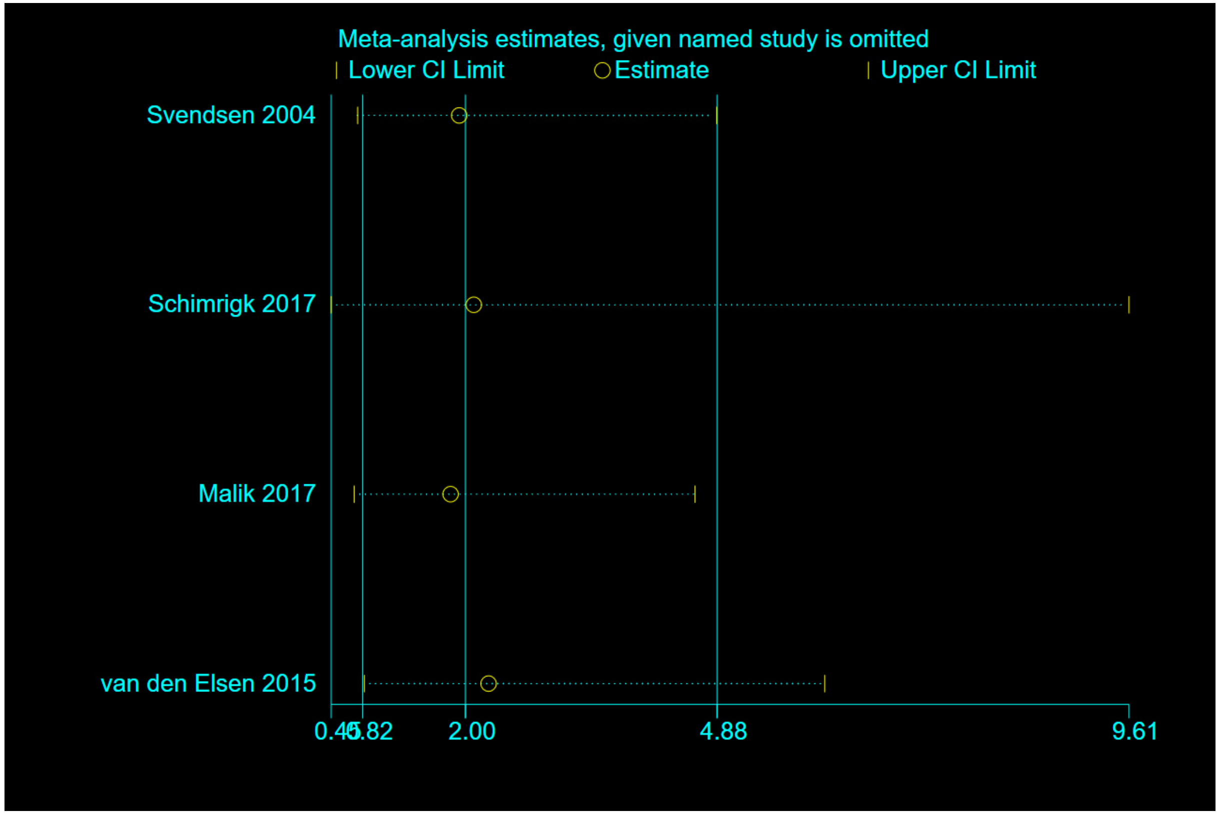

Meta-analysis estimates, given named study is omitted
Lower CI Limit
Estimate
Upper CI Limit
Svendsen 2004
Schimrigk 2017
Malik 2017
van den Elsen 2015
0.46
0.82
2.00
4.88
9.61


fatigue

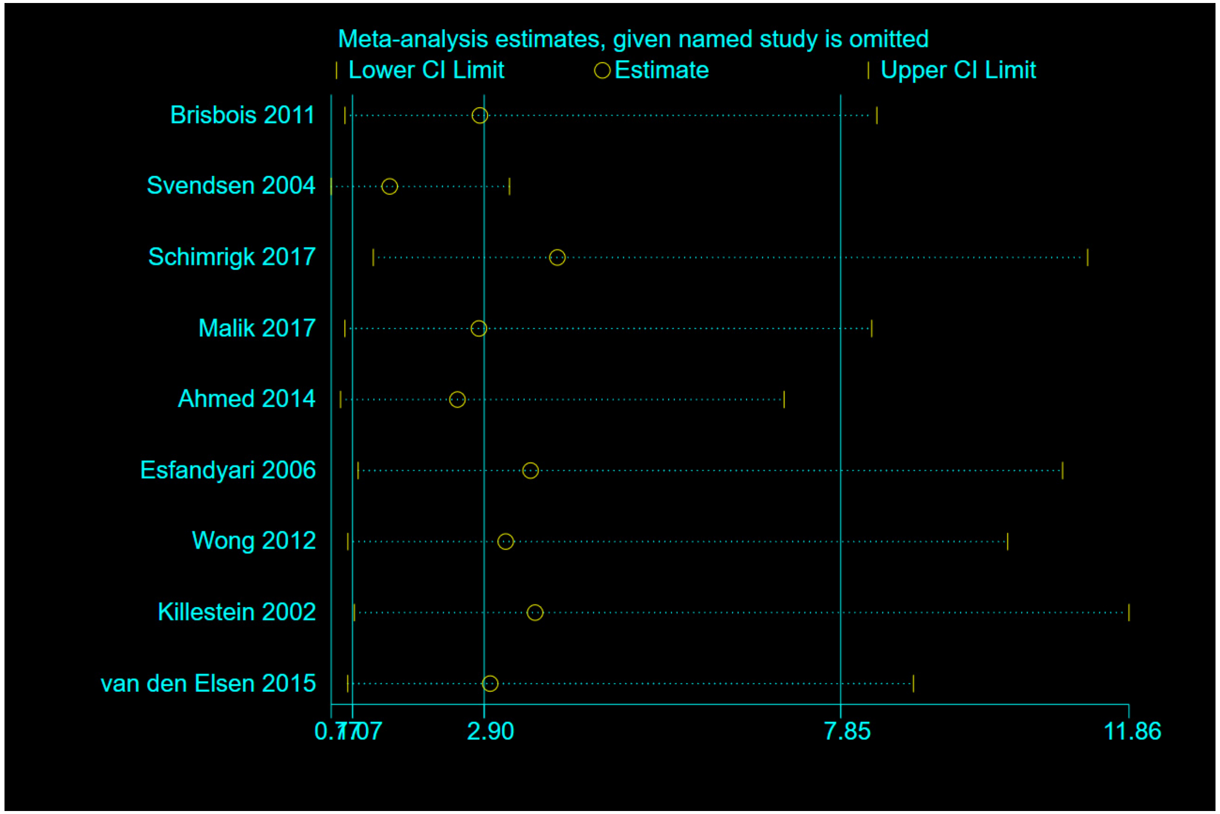
Meta-analysis estimates, given named study is omitted
Lower CI Limit
Estimate
Upper CI Limit
Brisbois 2011
Svendsen 2004
Schimrigk 2017
Malik 2017
Ahmed 2014
Esfandyari 2006
Wong 2012
Killestein 2002
van den Elsen 2015
0.7707
2.90
7.85
11.86

headache

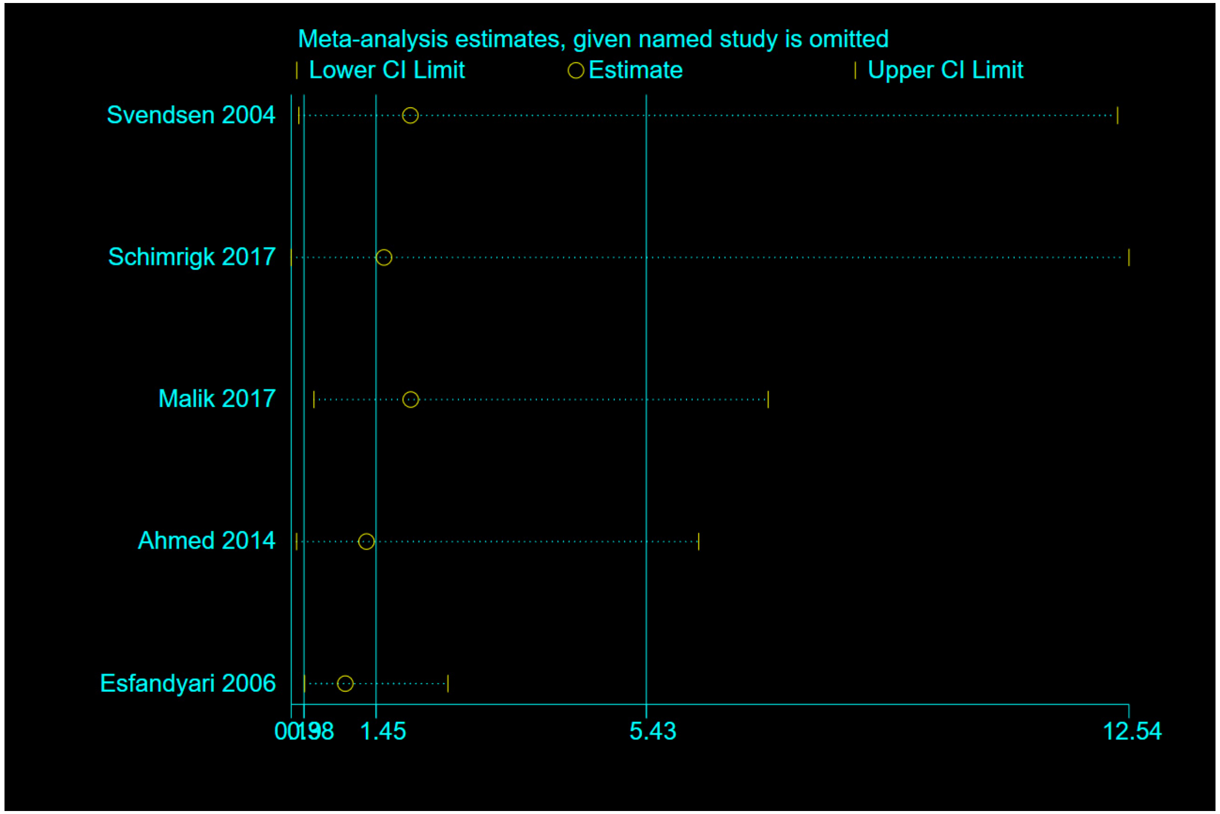
Meta-analysis estimates, given named study is omitted
Lower CI Limit
Estimate
Upper CI Limit
Svendsen 2004
Schimrigk 2017
Malik 2017
Ahmed 2014
Esfandyari 2006
1.45
5.43
12.54

nausea

**Table S1** PRISMA Checklist

| Section and Topic | Item # | Checklist item | Location where item is reported |
|---|---|---|---|
| **TITLE** | | | |
| Title | 1 | Identify the report as a systematic review. | page 1 |
| **ABSTRACT** | | | |
| Abstract | 2 | See the PRISMA 2020 for Abstracts checklist. | 2 |
| **INTRODUCTION** | | | |
| Rationale | 3 | Describe the rationale for the review in the context of existing knowledge. | 3 |
| Objectives | 4 | Provide an explicit statement of the objective(s) or question(s) the review addresses. | 3 |
| **METHODS** | | | |
| Eligibility criteria | 5 | Specify the inclusion and exclusion criteria for the review and how studies were grouped for the syntheses. | 4 |
| Information sources | 6 | Specify all databases, registers, websites, organisations, reference lists and other sources searched or consulted to identify studies. Specify the date when each source was last searched or consulted. | 4 |
| Search strategy | 7 | Present the full search strategies for all databases, registers and websites, including any filters and limits used. | 4 |
| Selection process | 8 | Specify the methods used to decide whether a study met the inclusion criteria of the review, including how many reviewers screened each record and each report retrieved, whether they worked independently, and if applicable, details of automation tools used in the process. | 4 |
| Data collection process | 9 | Specify the methods used to collect data from reports, including how many reviewers collected data from each report, whether they worked independently, any processes for obtaining or confirming data from study investigators, and if applicable, details of automation tools used in the process. | 4 |
| Data items | 10a | List and define all outcomes for which data were sought. Specify whether all results that were compatible with each outcome domain in each study were sought (e.g. for all measures, time points, analyses), and if not, the methods used to decide which results to collect. | 5 |
| | 10b | List and define all other variables for which data were sought (e.g. participant and intervention characteristics, funding sources). Describe any assumptions made about any missing or unclear information. | 5 |
| Study risk of bias assessment | 11 | Specify the methods used to assess risk of bias in the included studies, including details of the tool(s) used, how many reviewers assessed each study and whether they worked independently, and if applicable, details of automation tools used in the process. | 5 |
| Effect measures | 12 | Specify for each outcome the effect measure(s) (e.g. risk ratio, mean difference) used in the synthesis or presentation of results. | 5 |
| Synthesis methods | 13a | Describe the processes used to decide which studies were eligible for each synthesis (e.g. tabulating the study intervention characteristics and comparing against the planned groups for each synthesis (item #5)). | 5 |
| | 13b | Describe any methods required to prepare the data for presentation or synthesis, such as handling of missing summary statistics, or data conversions. | 5 |
| | 13c | Describe any methods used to tabulate or visually display results of individual studies and syntheses. | 5 |
| | 13d | Describe any methods used to synthesize results and provide a rationale for the choice(s). If meta-analysis was performed, describe the | 5 |

| Section and Topic | Item # | Checklist item | Location where item is reported |
|---|---|---|---|
| | | model(s), method(s) to identify the presence and extent of statistical heterogeneity, and software package(s) used. | |
| | 13e | Describe any methods used to explore possible causes of heterogeneity among study results (e.g. subgroup analysis, meta-regression). | 5 |
| | 13f | Describe any sensitivity analyses conducted to assess robustness of the synthesized results. | 5 |
| Reporting bias assessment | 14 | Describe any methods used to assess risk of bias due to missing results in a synthesis (arising from reporting biases). | 5 |
| Certainty assessment | 15 | Describe any methods used to assess certainty (or confidence) in the body of evidence for an outcome. | - |
| **RESULTS** | | | |
| Study selection | 16a | Describe the results of the search and selection process, from the number of records identified in the search to the number of studies included in the review, ideally using a flow diagram. | 6 |
| | 16b | Cite studies that might appear to meet the inclusion criteria, but which were excluded, and explain why they were excluded. | 6 |
| Study characteristics | 17 | Cite each included study and present its characteristics. | 6 |
| Risk of bias in studies | 18 | Present assessments of risk of bias for each included study. | 6 |
| Results of individual studies | 19 | For all outcomes, present, for each study: (a) summary statistics for each group (where appropriate) and (b) an effect estimate and its precision (e.g. confidence/credible interval), ideally using structured tables or plots. | 8-9 |
| Results of syntheses | 20a | For each synthesis, briefly summarise the characteristics and risk of bias among contributing studies. | 9-10 |
| | 20b | Present results of all statistical syntheses conducted. If meta-analysis was done, present for each the summary estimate and its precision (e.g. confidence/credible interval) and measures of statistical heterogeneity. If comparing groups, describe the direction of the effect. | 9-10 |
| | 20c | Present results of all investigations of possible causes of heterogeneity among study results. | 9-10 |
| | 20d | Present results of all sensitivity analyses conducted to assess the robustness of the synthesized results. | - |
| Reporting biases | 21 | Present assessments of risk of bias due to missing results (arising from reporting biases) for each synthesis assessed. | - |
| Certainty of evidence | 22 | Present assessments of certainty (or confidence) in the body of evidence for each outcome assessed. | - |
| **DISCUSSION** | | | |
| Discussion | 23a | Provide a general interpretation of the results in the context of other evidence. | 12-13 |
| | 23b | Discuss any limitations of the evidence included in the review. | 12-13 |
| | 23c | Discuss any limitations of the review processes used. | 12-13 |
| | 23d | Discuss implications of the results for practice, policy, and future research. | - |
| **OTHER INFORMATION** | | | |

| Section and Topic | Item # | Checklist item | Location where item is reported |
|---|---|---|---|
| Registration and protocol | 24a | Provide registration information for the review, including register name and registration number, or state that the review was not registered. | 4 |
| | 24b | Indicate where the review protocol can be accessed, or state that a protocol was not prepared. | 4 |
| | 24c | Describe and explain any amendments to information provided at registration or in the protocol. | - |
| Support | 25 | Describe sources of financial or non-financial support for the review, and the role of the funders or sponsors in the review. | 13 |
| Competing interests | 26 | Declare any competing interests of review authors. | 13 |
| Availability of data, code and other materials | 27 | Report which of the following are publicly available and where they can be found: template data collection forms; data extracted from included studies; data used for all analyses; analytic code; any other materials used in the review. | 14 |

*From:* Page MJ, McKenzie JE, Bossuyt PM, Boutron I, Hoffmann TC, Mulrow CD, et al. The PRISMA 2020 statement: an updated guideline for reporting systematic reviews. BMJ 2021;372:n71. doi: 10.1136/bmj.n71

For more information, visit: http://www.prisma-statement.org/

**Table S2**. List of excluded studies with the reason for exclusion

| | | |
|---|---|---|
| **Not clinical study** | 26 | Arn C, Weismann-Hügle T. Ethikwissen für Fachpersonen. 2009;(2). doi:10.1002/14651858.CD009464.pub2.www.cochranelibrary.com |
| | | Barnes MP. Sativex: clinical efficacy and tolerability in the treatment of symptoms of multiple sclerosis and neuropathic pain. Expert Opin Pharmacother. 2006;7(5):607-615. doi:10.1517/14656566.7.5.607 |
| | | Ben Amar M. Cannabinoids in medicine: A review of their therapeutic potential. J Ethnopharmacol. 2006;105(1-2):1-25. doi:10.1016/j.jep.2006.02.001 |
| | | Bhardwaj AK, Allsop DJ, Copeland J, et al. Randomised Controlled Trial (RCT) of cannabinoid replacement therapy (Nabiximols) for the management of treatment-resistant cannabis dependent patients: a study protocol. BMC Psychiatry. 2018;18(1):140. doi:10.1186/s12888-018-1682-2 |
| | | Borgelt LM, Franson KL, Nussbaum AM, Wang GS. The Pharmacologic and Clinical Effects of Medical Cannabis. Pharmacotherapy. 2013;33(2):195-209. doi:10.1002/phar.1187 |
| | | Boychuk DG, Goddard G, Mauro G, Orellana MF. The Effectiveness of Cannabinoids in the Management of Chronic Nonmalignant Neuropathic Pain: A Systematic Review. J ORAL FACIAL PAIN HEADACHE. 2015;29(1):7-14. doi:10.11607/ofph.1274 |
| | | da Rovare VP, Magalhaes GPA, Jardini GDA, et al. Cannabinoids for spasticity due to multiple sclerosis or paraplegia: A systematic review and meta-analysis of randomized clinical trials. Complement Ther Med. 2017;34:170-185. doi:10.1016/j.ctim.2017.08.010 |
| | | Fitzcharles M-A, Baerwald C, Ablin J, Haeuser W. Efficacy, tolerability and safety of cannabinoids in chronic pain associated with rheumatic diseases (fibromyalgia syndrome, back pain, osteoarthritis, rheumatoid arthritis) A systematic review of randomized controlled trials. SCHMERZ. 2016;30(1):47-61. doi:10.1007/s00482-015-0084-3 |
| | | Fitzcharles M-A, Ste-Marie PA, Haeuser W, et al. Efficacy, Tolerability, and Safety of Cannabinoid Treatments in the Rheumatic Diseases: A Systematic Review of Randomized Controlled Trials. Arthritis Care Res (Hoboken). 2016;68(5):681-688. doi:10.1002/acr.22727 |
| | | Hindocha C, Cousijn J, Rall M, Bloomfield MAP. The Effectiveness of Cannabinoids in the Treatment of Posttraumatic Stress Disorder (PTSD): A Systematic Review. J Dual Diagn. 2020;16(1):120-139. doi:10.1080/15504263.2019.1652380 |
| | | J. A, G. S-L, Aviram J, Samuelly-Leichtag G. Efficacy of cannabis-based medicines for pain management: A systematic review and meta-analysis of randomized controlled trials. Pain Physician. 2017;20(6):E755-E796. http://www.embase.com/search/results?subaction=viewrecord&from=export&id=L618450120. |
| | | Lim K, See YM, Lee J, K. L, Y.M. S, J. L. A systematic review of the effectiveness of medical cannabis for psychiatric, movement and neurodegenerative disorders. Clin Psychopharmacol Neurosci. 2017;15(4):301-312. doi:10.9758/cpn.2017.15.4.301 |
| | | Lynch ME, Ware MA. Cannabinoids for the Treatment of Chronic Non-Cancer Pain: An Updated Systematic Review of Randomized Controlled Trials. J NEUROIMMUNE Pharmacol. 2015;10(2):293-301. doi:10.1007/s11481-015-9600-6 |

| | | |
|---|---|---|
| | | Lynch ME, Campbell F. Cannabinoids for treatment of chronic non-cancer pain; a systematic review of randomized trials. Br J Clin Pharmacol. 2011;72(5):735-744. doi:10.1111/j.1365-2125.2011.03970.x |
| | | M.A. W, G.T. K, A.G. de A, et al. A Systematic Review of the Efficacy of Cannabinoid Agonist Replacement Therapy for Cannabis Withdrawal Symptoms. CNS Drugs. 2018;32(12):1113-1129. doi:10.1007/s40263-018-0577-6 |
| | | Machado Rocha FC, Stefano SC, De Cassia Haiek R, Rosa Oliveira LMQ, Da Silveira DX. Therapeutic use of Cannabis sativa on chemotherapy-induced nausea and vomiting among cancer patients: systematic review and meta-analysis. Eur J Cancer Care (Engl). 2008;17(5):431-443. doi:10.1111/j.1365-2354.2008.00917.x |
| | | Meng H, Johnston B, Englesakis M, Moulin DE, Bhatia A. Selective Cannabinoids for Chronic Neuropathic Pain: A Systematic Review and Meta-analysis. Anesth Analg. 2017;125(5):1638-1652. doi:10.1213/ANE.0000000000002110 |
| | | Moreno Torres I, Sanchez AJ, Garcia-Merino A. Evaluation of the tolerability and efficacy of Sativex in multiple sclerosis. Expert Rev Neurother. 2014;14(11):1243-1250. doi:10.1586/14737175.2014.971758 |
| | | Sastre-Garriga J, Vila C, Clissold S, Montalban X. THC and CBD oromucosal spray (Sativex(R)) in the management of spasticity associated with multiple sclerosis. Expert Rev Neurother. 2011;11(5):627-637. doi:10.1586/ern.11.47 |
| | | Syed YY, McKeage K, Scott LJ. Delta-9-tetrahydrocannabinol/cannabidiol (Sativex(R)): a review of its use in patients with moderate to severe spasticity due to multiple sclerosis. Drugs. 2014;74(5):563-578. doi:10.1007/s40265-014-0197-5 |
| | | Tafelski S, Häuser W, Schäfer M. Wirksamkeit, Verträglichkeit und Sicherheit von Cannabinoiden für die Therapie von Chemotherapie-induzierter Übelkeit und Erbrechen: eine systematische Zusammenfassung systematischer Reviews. Schmerz. 2016;30(1):14-24. doi:10.1007/s00482-015-0092-3 |
| | | Tsang CC, Giudice MG. Nabilone for the Management of Pain. Pharmacotherapy. 2016;36(3):273-286. doi:10.1002/phar.1709 |
| | | van den Elsen GAH, Ahmed AIA, Lammers M, et al. Efficacy and safety of medical cannabinoids in older subjects: A systematic review. AGEING Res Rev. 2014;14:56-64. doi:10.1016/j.arr.2014.01.007 |
| | | Vermersch P. Sativex((R)) (tetrahydrocannabinol + cannabidiol), an endocannabinoid system modulator: basic features and main clinical data. Expert Rev Neurother. 2011;11(4 Suppl):15-19. doi:10.1586/ern.11.27 |
| | | Wade DT, Collin C, Stott C, Duncombe P. Meta-analysis of the efficacy and safety of Sativex (nabiximols), on spasticity in people with multiple sclerosis. Mult Scler. 2010;16(6):707-714. doi:10.1177/1352458510367462 |
| | | Whiting PF, Wolff RF, Deshpande S, et al. Cannabinoids for medical use: A systematic review and meta-analysis. JAMA - J Am Med Assoc. 2015;313(24):2456-2473. doi:10.1001/jama.2015.6358 |
| **Not placebo-controlled setting** | 22 | Beaulieu P. Effects of nabilone, a synthetic cannabinoid, on postoperative pain. Can J Anesth. 2006;53(8):769-775. doi:10.1007/BF03022793 |
| | | Bisaga A, Sullivan MA, Glass A, et al. The effects of dronabinol during detoxification and the initiation of treatment with extended release naltrexone. Drug Alcohol Depend. 2015;154:38-45. doi:10.1016/j.drugalcdep.2015.05.013 |
| | | Chang AE, Shiling DJ, Stillman RC, et al. Delata-9-tetrahydrocannabinol as an antiemetic in cancer patients receiving high-dose methotrexate. A prospective, randomized evaluation. Ann Intern Med. 1979;91(6):819-824. doi:10.7326/0003-4819-91-6-819 |

| | | |
|---|---|---|
| | | Cunningham D, Bradley CJ, Forrest GJ, et al. A randomized trial of oral nabilone and prochlorperazine compared to intravenous metoclopramide and dexamethasone in the treatment of nausea and vomiting induced by chemotherapy regimens containing cisplatin or cisplatin analogues. Eur J Cancer Clin Oncol. 1988;24(4):685-689. doi:10.1016/0277-5379(88)90300-8 |
| | | de Vries M, van Rijckevorsel DCM, Vissers KCP, Wilder-Smith OHG, van Goor H. Single dose delta-9-tetrahydrocannabinol in chronic pancreatitis patients: analgesic efficacy, pharmacokinetics and tolerability. Br J Clin Pharmacol. 2016;81(3, SI):525-537. doi:10.1111/bcp.12811 |
| | | Ellis RJ, Toperoff W, Vaida F, et al. Smoked medicinal cannabis for neuropathic pain in HIV: a randomized, crossover clinical trial. Neuropsychopharmacology. 2009;34(3):672-680. doi:10.1038/npp.2008.120 |
| | | Frank B, Serpell MG, Hughes J, Matthews JNS, Kapur D. Comparison of analgesic effects and patient tolerability of nabilone and dihydrocodeine for chronic neuropathic pain: randomised, crossover, double blind study. Br Med J. 2008;336(7637):199-201. doi:10.1136/bmj.39429.619653.80 |
| | | Gilbert CJ, Ohly K V., Rosner G, Peters WP. Randomized, double-blind comparison of a prochlorperazine-based versus a metoclopramide-based antiemetic regimen in patients undergoing autologous bone marrow transplantation. Cancer. 1995;76(11):2330-2337. doi:10.1002/1097-0142(19951201)76:11<2330::AID-CNCR2820761122>3.0.CO;2-F |
| | | Johansson R. A d o u b l e - b l l n d , c o n t r o l l e d t r i a l o f n a b H o n e vs . procldorperazine for refractory emesis induced by cancer chemotherapy. 1982;9:25-33. |
| | | Kleine-Brueggeney M, Greif R, Brenneisen R, Urwyler N, Stueber F, Theiler LG. Intravenous Delta-9-Tetrahydrocannabinol to Prevent Postoperative Nausea and Vomiting: A Randomized Controlled Trial. Anesth Analg. 2015;121(5):1157-1164. doi:10.1213/ANE.0000000000000877 |
| | | Knoller N, Levi L, Shoshan I, et al. Dexanabinol (HU-211) in the treatment of severe closed head injury: a randomized, placebo-controlled, phase II clinical trial. Crit Care Med. 2002;30(3):548-554. doi:10.1097/00003246-200203000-00009 |
| | | Levin FR, Mariani JJ, Brooks DJ, Pavlicova M, Cheng W, Nunes E V. Dronabinol for the treatment of cannabis dependence: A randomized, double-blind, placebo-controlled trial. Drug Alcohol Depend. 2011;116(1-3):142-150. doi:10.1016/j.drugalcdep.2010.12.010 |
| | | Levin FR, Mariani JJ, Pavlicova M, et al. Dronabinol and lofexidine for cannabis use disorder: A randomized, double-blind, placebo-controlled trial. Drug Alcohol Depend. 2016;159:53-60. doi:10.1016/j.drugalcdep.2015.11.025 |
| | | Maas AIR, Murray G, Henney H 3rd, et al. Efficacy and safety of dexanabinol in severe traumatic brain injury: results of a phase III randomised, placebo-controlled, clinical trial. Lancet Neurol. 2006;5(1):38-45. doi:10.1016/S1474-4422(05)70253-2 |
| | | Micallef J, Dupouey J, Jouve E, et al. Cannabis smoking impairs driving performance on the simulator and real driving: a randomized, double-blind, placebo-controlled, crossover trial. Fundam Clin Pharmacol. 2018;32(5):558-570. doi:10.1111/fcp.12382 |
| | | Pini LA, Guerzoni S, Cainazzo MM, et al. Nabilone for the treatment of medication overuse headache: results of a preliminary double-blind, active-controlled, randomized trial. J Headache Pain. 2012;13(8):677-684. doi:10.1007/s10194-012-0490-1 |

| | | |
|---|---|---|
| | | Priestman SG, Priestman TJ, Canney PA. A double-blind randomised cross-over comparison of nabilone and metoclopramide in the control of radiation-induced nausea. Clin Radiol. 1987;38(5):543-544. doi:10.1016/s0009-9260(87)80151-4 |
| | | Rintala DH, Fiess RN, Tan G, Holmes SA, Bruel BM. Effect of Dronabinol on Central Neuropathic Pain After Spinal Cord Injury. Am J Phys Med Rehabil. 2010;89(10):840-848. doi:10.1097/PHM.0b013e3181f1c4ec |
| | | Turcotte D, Doupe M, Torabi M, et al. Nabilone as an adjunctive to gabapentin for multiple sclerosis-induced neuropathic pain: A randomized controlled trial. Pain Med (United States). 2015;16(1):149-159. doi:10.1111/pme.12569 |
| | | van de Donk T, Niesters M, Kowal MA, Olofsen E, Dahan A, van Velzen M. An experimental randomized study on the analgesic effects of pharmaceutical-grade cannabis in chronic pain patients with fibromyalgia. Pain. 2019;160(4):860-869. doi:10.1097/j.pain.0000000000001464 |
| | | Wallace MS, Marcotte TD, Umlauf A, Gouaux B, Atkinson JH. Efficacy of Inhaled Cannabis on Painful Diabetic Neuropathy. J Pain. 2015;16(7):616-627. doi:10.1016/j.jpain.2015.03.008 |
| | | Ware MA, Fitzcharles M-A, Joseph L, Shir Y. The Effects of Nabilone on Sleep in Fibromyalgia: Results of a Randomized Controlled Trial. Anesth Analg. 2010;110(2):604-610. doi:10.1213/ANE.0b013e3181c76f70 |
| **Missing or inappropriate data** | 15 | Ball S, Vickery J, Hobart J, et al. The Cannabinoid Use in Progressive Inflammatory brain Disease (CUPID) trial: a randomised double-blind placebo-controlled parallel-group multicentre trial and economic evaluation of cannabinoids to slow progression in multiple sclerosis. Health Technol Assess. 2015;19(12):vii-viii, xxv-xxxi, 1-187. doi:10.3310/hta19120 |
| | | Barton C, Sklenicka J, Sayegh P, et al. A double-blind trial of bupropion versus desipramine for bipolar depression. J Clin Psychiatry. 2003;55(2):391-393. doi:10.1002/hup |
| | | Crawford SM, Buckman R. Nabilone and metoclopramide in the treatment of nausea and vomiting due to cisplatinum: A double blind study. Med Oncol Tumor Pharmacother. 1986;3(1):39-42. doi:10.1007/BF02934575 |
| | | Fox SH, Kellett M, Moore AP, Crossman AR, Brotchie JM. Randomised, double-blind, placebo-controlled trial to assess the potential of cannabinoid receptor stimulation in the treatment of dystonia. Mov Disord. 2002;17(1):145-149. doi:10.1002/mds.1280 |
| | | Jadoon KA, Ratcliffe SH, Barrett DA, et al. Efficacy and Safety of Cannabidiol and Tetrahydrocannabivarin on Glycemic and Lipid Parameters in Patients With Type 2 Diabetes: A Randomized, Double-Blind, Placebo-Controlled, Parallel Group Pilot Study. Diabetes Care. 2016;39(10):1777-1786. doi:10.2337/dc16-0650 |
| | | Jatoi A, Windschitl HE, Loprinzi CL, et al. Dronabinol versus megestrol acetate versus combination therapy for cancer-associated anorexia: A North Central Cancer Treatment Group Study. J Clin Oncol. 2002;20(2):567-573. doi:10.1200/JCO.20.2.567 |
| | | Klumpers LE, Beumer TL, van Hasselt JGC, et al. Novel Delta(9)-tetrahydrocannabinol formulation Namisol (R) has beneficial pharmacokinetics and promising pharmacodynamic effects. Br J Clin Pharmacol. 2012;74(1):42-53. doi:10.1111/j.1365-2125.2012.04164.x |
| | | Levin DN, Dulberg Z, Chan AW, Hare GMT, Mazer CD, Hong A. Une étude randomisée contrôlée pour évaluer l’efficacité du nabilone pour la prévention des nausées et vomissements postopératoires aigus lors de chirurgie non urgente. Can J Anesth. 2017;64(4):385-395. doi:10.1007/s12630-017-0814-3 |

| | | |
|---|---|---|
| | | Lynch ME, Cesar-Rittenberg P, Hohmann AG. A double-blind, placebo-controlled, crossover pilot trial with extension using an oral mucosal cannabinoid extract for treatment of chemotherapy-induced neuropathic pain. J Pain Symptom Manage. 2014;47(1):166-173. doi:10.1016/j.jpainsymman.2013.02.018 |
| | | Markovà J, Essner U, Akmaz B, et al. Sativex ® as add-on therapy vs. further optimized first-line ANTispastics (SAVANT) in resistant multiple sclerosis spasticity: a double-blind, placebo-controlled randomised clinical trial. Int J Neurosci. 2019;129(2):119-128. doi:10.1080/00207454.2018.1481066 |
| | | Strasser F, Luftner D, Possinger K, et al. Comparison of orally administered cannabis extract and delta-9-tetrahydrocannabinol in treating patients with cancer-related anorexia-cachexia syndrome: A multicenter, phase III, randomized, double-blind, placebo-controlled clinical trial from the cannabi. J Clin Oncol. 2006;24(21):3394-3400. doi:10.1200/JCO.2005.05.1847 |
| | | van den Elsen GAH, Ahmed AIA, Verkes R-J, Feuth T, van der Marck MA, Olde Rikkert MGM. Tetrahydrocannabinol in Behavioral Disturbances in Dementia: A Crossover Randomized Controlled Trial. Am J Geriatr Psychiatry. 2015;23(12):1214-1224. doi:10.1016/j.jagp.2015.07.011 |
| | | van den Elsen GA, Tobben L, Ahmed AI, et al. Effects of tetrahydrocannabinol on balance and gait in patients with dementia: A randomised controlled crossover trial. J Psychopharmacol. 2017;31(2):184-191. doi:10.1177/0269881116665357 |
| | | Walter C, Oertel BG, Ludyga D, Ultsch A, Hummel T, Lotsch J. Effects of 20 mg oral Delta(9) -tetrahydrocannabinol on the olfactory function of healthy volunteers. Br J Clin Pharmacol. 2014;78(5):961-969. doi:10.1111/bcp.12415 |
| | | Zajicek J, Ball S, Wright D, et al. Effect of dronabinol on progression in progressive multiple sclerosis (CUPID): a randomised, placebo-controlled trial. LANCET Neurol. 2013;12(9):857-865. doi:10.1016/S1474-4422(13)70159-5 |
| **Other study drug than nabilone or dronabinol** | 22 | Aragona M, Onesti E, Tomassini V, et al. Psychopathological and cognitive effects of therapeutic cannabinoids in multiple clerosis: A double-blind, placebo controlled, crossover study. Clin Neuropharmacol. 2009;32(1):41-47. doi:10.1097/WNF.0b013e3181633497 |
| | | Berman JS, Symonds C, Birch R. Efficacy of two cannabis based medicinal extracts for relief of central neuropathic pain from brachial plexus avulsion: results of a randomised controlled trial. Pain. 2004;112(3):299-306. doi:10.1016/j.pain.2004.09.013 |
| | | Collin C, Davies P, Mutiboko IK, Ratcliffe S. Randomized controlled trial of cannabis-based medicine in spasticity caused by multiple sclerosis. Eur J Neurol. 2007;14(3):290-296. doi:10.1111/j.1468-1331.2006.01639.x |
| | | Collin C, Ehler E, Waberzinek G, et al. A double-blind, randomized, placebo-controlled, parallel-group study of Sativex, in subjects with symptoms of spasticity due to multiple sclerosis. Neurol Res. 2010;32(5):451-459. doi:10.1179/016164109X12590518685660 |
| | | Conte A, Bettolo CM, Onesti E, et al. Cannabinoid-induced effects on the nociceptive system: A neurophysiological study in patients with secondary progressive multiple sclerosis. Eur J Pain. 2009;13(5):472-477. doi:10.1016/j.ejpain.2008.05.014 |
| | | D.J. R, T.J. N, T. F, et al. Randomized, controlled trial of cannabis-based medicine in central pain in multiple sclerosis. Neurology. 2005;65(6):812-819. doi:10.1212/01.wnl.0000176753.45410.8b |
| | | Duran M, Pérez E, Abanades S, et al. Preliminary efficacy and safety of an oromucosal standardized cannabis extract in chemotherapy-induced nausea and vomiting. Br J Clin Pharmacol. 2010;70(5):656-663. doi:10.1111/j.1365-2125.2010.03743.x |

| | | |
|---|---|---|
| | | Johnson JR, Burnell-Nugent M, Lossignol D, Ganae-Motan ED, Potts R, Fallon MT. Multicenter, Double-Blind, Randomized, Placebo-Controlled, Parallel-Group Study of the Efficacy, Safety, and Tolerability of THC:CBD Extract and THC Extract in Patients with Intractable Cancer-Related Pain. J Pain Symptom Manage. 2010;39(2):167-179. doi:10.1016/j.jpainsymman.2009.06.008 |
| | | Kavia RBC, De Ridder D, Constantinescu CS, Stott CG, Fowler CJ. Randomized controlled trial of Sativex to treat detrusor overactivity in multiple sclerosis. Mult Scler. 2010;16(11):1349-1359. doi:10.1177/1352458510378020 |
| | | Langford RM, Mares J, Novotna A, et al. A double-blind, randomized, placebo-controlled, parallel-group study of THC/CBD oromucosal spray in combination with the existing treatment regimen, in the relief of central neuropathic pain in patients with multiple sclerosis. J Neurol. 2013;260(4):984-997. doi:10.1007/s00415-012-6739-4 |
| | | Leocani L, Nuara A, Houdayer E, et al. Sativex® and clinical–neurophysiological measures of spasticity in progressive multiple sclerosis. J Neurol. 2015;262(11):2520-2527. doi:10.1007/s00415-015-7878-1 |
| | | Lichtman AH, Lux EA, McQuade R, et al. Results of a Double-Blind, Randomized, Placebo-Controlled Study of Nabiximols Oromucosal Spray as an Adjunctive Therapy in Advanced Cancer Patients with Chronic Uncontrolled Pain. J Pain Symptom Manage. 2018;55(2):179-188.e1. doi:10.1016/j.jpainsymman.2017.09.001 |
| | | López-Sendón Moreno JL, García Caldentey J, Trigo Cubillo P, et al. A double-blind, randomized, cross-over, placebo-controlled, pilot trial with Sativex in Huntington's disease. J Neurol. 2016;263(7):1390-1400. doi:10.1007/s00415-016-8145-9 |
| | | Notcutt W, Langford R, Davies P, Ratcliffe S, Potts R. A placebo-controlled, parallel-group, randomized withdrawal study of subjects with symptoms of spasticity due to multiple sclerosis who are receiving long-term Sativex (nabiximols). Mult Scler J. 2012;18(2):219-228. doi:10.1177/1352458511419700 |
| | | Novotna A, Mares J, Ratcliffe S, et al. A randomized, double-blind, placebo-controlled, parallel-group, enriched-design study of nabiximols* (Sativex((R)) ), as add-on therapy, in subjects with refractory spasticity caused by multiple sclerosis. Eur J Neurol. 2011;18(9):1122-1131. doi:10.1111/j.1468-1331.2010.03328.x |
| | | Nurmikko TJ, Serpell MG, Hoggart B, Toomey PJ, Morlion BJ, Haines D. Sativex successfully treats neuropathic pain characterised by allodynia: a randomised, double-blind, placebo-controlled clinical trial. Pain. 2007;133(1-3):210-220. doi:10.1016/j.pain.2007.08.028 |
| | | Portenoy RK, Ganae-Motan ED, Allende S, et al. Nabiximols for opioid-treated cancer patients with poorly-controlled chronic pain: A randomized, placebo-controlled, graded-dose trial. J Pain. 2012;13(5):438-449. doi:10.1016/j.jpain.2012.01.003 |
| | | Serpell M, Ratcliffe S, Hovorka J, et al. A double-blind, randomized, placebo-controlled, parallel group study of THC/CBD spray in peripheral neuropathic pain treatment. Eur J Pain (United Kingdom). 2014;18(7):999-1012. doi:10.1002/j.1532-2149.2013.00445.x |
| | | Vaney C, Heinzel-Gutenbrunner M, Jobin P, et al. Efficacy, safety and tolerability of an orally administered cannabis extract in the treatment of spasticity in patients with multiple sclerosis: a randomized, double-blind, placebo-controlled, crossover study. Mult Scler. 2004;10(4):417-424. doi:10.1191/1352458504ms1048oa |
| | | Wade DT, Makela P, Robson P, House H, Bateman C. Do cannabis-based medicinal extracts have general or specific effects on symptoms in multiple sclerosis? A double-blind, randomized, placebo-controlled study on 160 patients. Mult Scler. 2004;10(4):434-441. doi:10.1191/1352458504ms1082oa |

| | | |
|---|---|---|
| | | Wade DT, Robson P, House H, Makela P, Aram J. A preliminary controlled study to determine whether whole-plant cannabis extracts can improve intractable neurogenic symptoms. Clin Rehabil. 2003;17(1):21-29. doi:10.1191/0269215503cr581oa |
| | | Ware MA, Wang T, Shapiro S, et al. Smoked cannabis for chronic neuropathic pain: a randomized controlled trial. CMAJ. 2010;182(14):E694-701. doi:10.1503/cmaj.091414 |

**Table S3**. Adverse events in the clinical trials with nabilone

| Adverse effects (nabilone) | ICD | No. of studies reporting the AE | References | Classification |
|---|---|---|---|---|
| Ataxia | R2700 | 2 | Pooyania, 2010<br>Skrabek, 2008 | Central nervous system |
| Confusion | R4100 | 1 | Skrabek, 2008 | Central nervous system |
| Decreased concentration | F9900 | 1 | Skrabek, 2008 | Central nervous system |
| Depression | F32H0 | 1 | Skrabek, 2008 | Central nervous system |
| Dissociation | F44H0 | 1 | Skrabek, 2008 | Central nervous system |
| Euphoria | F31H0 | 2 | Redmond, 2008<br>Skrabek, 2008 | Central nervous system |
| Feeling cold | F3800 | 1 | Redmond, 2008 | Central nervous system |
| Hallucination | R4430 | x | Skrabek, 2008 | Central nervous system |
| Mild sedation | F1310 | 1 | Redmond, 2008 | Central nervous system |
| Nightmares | F5150 | 1 | Skrabek, 2008 | Central nervous system |
| Psychological high | F3800 | 1 | Skrabek, 2008 | Central nervous system |
| Sedation (Including Lethargy) | F1310 | 1 | Hermann, 2019 | Central nervous system |
| Significant increase in NPS | R7490 | 1 | Hermann, 2019 | Central nervous system |
| Somnolence | R4000 | 1 | Kalliomäki 2012 | Central nervous system |
| Treatment limiting sedation | F1310 | 1 | Hermann, 2019 | Central nervous system |
| Bradycardia | R0010 | 2 | Hermann, 2019<br>Kalliomäki 2012 | Cardiovascular |
| Myocardial infarction | I2100 | 1 | Hermann, 2019 | Cardiovascular |
| Orthostatic hypotension | I9510 | 1 | Skrabek, 2008 | Cardiovascular |
| Postural dizziness | I9510 | 1 | Kalliomäki 2012 | Cardiovascular |
| Tachycardia | R0000 | 2 | Kalliomäki 2012<br>Skrabek, 2008 | Cardiovascular |
| Anorexia | R6300 | 1 | Skrabek, 2008 | Miscellaneous |

| | | | | |
|---|---|---|---|---|
| Asthenia | R5300 | 1 | Pooyania, 2010 | Miscellaneous |
| Dizziness | R4200 | 3 | Hermann, 2019<br>Kalliomäki 2012<br>Redmond 2008 | Miscellaneous |
| Drowsiness | R4000 | 3 | Pooyania, 2010<br>Wissel 2006<br>Skrabek, 2008 | Miscellaneous |
| Dry mouth | R6820 | 4 | Pooyania, 2010<br>Kalliomäki 2012<br>Redmond, 2008<br>Skrabek, 2008 | Miscellaneous |
| Dysphagia (slight) | R13H0 | 1 | Wissel 2006 | Miscellaneous |
| Dysphoria | R53H0 | 1 | Skrabek, 2008 | Miscellaneous |
| Fatigue | R5300 | 1 | Kalliomäki 2012 | Miscellaneous |
| Headache | R5100 | 4 | Pooyania, 2010<br>Kalliomäki 2012<br>Redmond, 2008<br>Skrabek, 2008 | Miscellaneous |
| Increased appetite | R6320 | 1 | Redmond, 2008 | Miscellaneous |
| Lack of motivation | R4530 | 1 | Pooyania, 2010 | Miscellaneous |
| Lethargy | R5300 | 1 | Hermann, 2019 | Miscellaneous |
| Lightheaded | R4200 | 1 | Skrabek, 2008 | Miscellaneous |
| Nausea | R13H0 | 1 | Redmond, 2008 | Miscellaneous |
| Rash | R2100 | 1 | Hermann, 2019 | Miscellaneous |
| Sensory disturbance | R2000 | 1 | Skrabek, 2008 | Miscellaneous |
| Vertigo | R42H0 | 1 | Skrabek, 2008 | Miscellaneous |
| Vertigo (mild) | R42H0 | 1 | Pooyania, 2010 | Miscellaneous |
| Weakness in lower limbs (slight) | R6880 | 1 | Wissel 2006 | Miscellaneous |

**Table S4**. Adverse events in the clinical trials with dronabinol

| Adverse events (dronabinol) | ICD | No. of studies reporting the AE | Reference | Classification |
|---|---|---|---|---|
| "Loopy", foggy thinking | F3800 | 1 | Wong, 2012 | Central nervous system |
| Agitation | R4510 | 1 | van den Elsen, 2015 | Central nervous system |
| Ataxia | R2700 | 1 | Killestein, 2002 | Central nervous system |
| Bradykinesia | F4440 | 1 | van den Elsen, 2015 | Central nervous system |
| Cognitive disorder | F0670 | 1 | van den Elsen, 2015 | Central nervous system |
| Concentration problem | F9900 | 1 | Ahmed, 2014 | Central nervous system |
| Confusion | R4100 | 1 | Brisbois, 2011 | Central nervous system |
| Delirium | F0580 | 1 | van den Elsen, 2015 | Central nervous system |
| Disturbed mental concentration | F9900 | 1 | Esfandyari, 2006 | Central nervous system |
| Emotional lability | F6030 | 1 | Killestein, 2002 | Central nervous system |
| Euphoria | F31H0 | 2 | Svendsen, 2004<br>Ahmed, 2014 | Central nervous system |
| Euphoria/relaxed | F31H0 | 1 | Esfandyari, 2006 | Central nervous system |
| Euphoric mood | F31H0 | 1 | van den Elsen, 2015 | Central nervous system |
| Excitement | F3090 | 1 | Esfandyari, 2006 | Central nervous system |
| Feeling of drunkenness | F3800 | 1 | Svendsen, 2004 | Central nervous system |
| Hyperactivity | F9000 | 1 | Svendsen, 2004 | Central nervous system |
| Insomnia | G4700 | 1 | Schimrigk, 2017 | Central nervous system |
| Migraine | G4300 | 1 | Svendsen, 2004 | Central nervous system |
| Multiple sclerosis aggravated | G3500 | 1 | Svendsen, 2004 | Central nervous system |
| Nervousness | R4500 | 1 | Svendsen, 2004 | Central nervous system |
| Relaxation | R5300 | 1 | Ahmed, 2014 | Central nervous system |
| Seizure | R5680 | 1 | Brisbois, 2011 | Central nervous system |
| Sleep difficulty | G4790 | 1 | Svendsen, 2004 | Central nervous system |

| | | | | |
|---|---|---|---|---|
| Somnolence | R4000 | 2 | Killestein, 2002<br>van den Elsen, 2015 | **Central nervous system** |
| Speech disorders | F8010 | 1 | Svendsen, 2004 | **Central nervous system** |
| Troubles sleeping | G4790 | 1 | Brisbois, 2011 | **Central nervous system** |
| Visual hallucinations | R4410 | 1 | Ahmed, 2014 | **Central nervous system** |
| Bowel obstruction / constipation | K5660/K5900 | 1 | Brisbois, 2011 | **Gastrointestinal** |
| Diarrhoea | K5910 | 1 | Brisbois, 2011 | **Gastrointestinal** |
| Lose stools | K5910 | 1 | Malik, 2017 | **Gastrointestinal** |
| Stomach cramps | K3180 | 1 | Brisbois, 2011 | **Gastrointestinal** |
| Thrush | K1370 | 1 | Brisbois, 2011 | **Gastrointestinal** |
| Abdominal pain | R1040 | 1 | Svenden, 2004 | **Miscellaneous** |
| Anorexia | R6300 | 1 | Svenden, 2004 | **Miscellaneous** |
| Aphasia | R4700 | 1 | van den Elsen, 2015 | **Miscellaneous** |
| Apraxia | R4820 | 1 | van den Elsen, 2015 | **Miscellaneous** |
| Balance difficulty | H8190 | 1 | Svenden, 2004 | **Miscellaneous** |
| Balance disorder | H8190 | 1 | van den Elsen, 2015 | **Miscellaneous** |
| Bladder | N3280 | 1 | Zajicek, 2003 | **Miscellaneous** |
| Blurred vision | H5380 | 1 | Ahmed, 2014 | **Miscellaneous** |
| Chest pain | R0730 | 1 | van den Elsen, 2015 | **Miscellaneous** |
| Chills | R6880/R5500 | 1 | Svenden, 2004 | **Miscellaneous** |
| Chronic obstructive pulmonary diease | J4490 | 1 | van den Elsen, 2015 | **Miscellaneous** |
| Coordination disturbance | R2780 | 1 | Ahmed, 2014 | **Miscellaneous** |
| Decreased appetite | R6330 | 1 | van den Elsen, 2015 | **Miscellaneous** |
| Depression or anxiety | F32H0 | 1 | Zajicek, 2003 | **Miscellaneous** |
| Diplopia | H5320 | 1 | Svenden, 2004 | **Miscellaneous** |

| | | | | |
|---|---|---|---|---|
| Dizziness | R4200 | 5 | Svenden, 2004<br>Schimrigk, 2017<br>Ahmed, 2014<br>Killestein, 2002<br>van den Elsen, 2015 | Miscellaneous |
| Dizziness/lightheadness | R4200 | 2 | Esfandyari, 2006<br>Wong, 2002 | Miscellaneous |
| Dizzy of lightheadedness | R4200 | 1 | Zajicek, 2003 | Miscellaneous |
| Drowsiness | R4000 | 3 | Svendsen, 2004<br>Ahmed, 2014<br>Esfandyari, 2006 | Miscellaneous |
| Drowsiness/tiredness | R4000 | 1 | Wong, 2012 | Miscellaneous |
| Dry eye | H0410 | 2 | Ahmed, 2014<br>van den Elsen, 2015 | Miscellaneous |
| Dry mouth | R6820 | 6 | Svendsen, 2004<br>Schimrigk, 2017<br>Ahmed, 2014<br>Esfandyari, 2006<br>Killestein 2002<br>Zajicek, 2003 | Miscellaneous |
| Edema | R6090 | 1 | Brisbois, 2011 | Miscellaneous |
| Eye hemorrhage | H4480 | 1 | van den Elsen, 2015 | Miscellaneous |
| Fatigue | R5300 | 4 | Svendsen, 2004<br>Schimrigk, 2017<br>Malik, 2017<br>van den Elsen, 2015 | Miscellaneous |
| Fever | R5090 | 2 | Brisbois, 2011<br>Svendsen, 2004 | Miscellaneous |
| Flushing | R3200 | 1 | Esfandyari, 2006 | Miscellaneous |

| | | | | |
|---|---|---|---|---|
| Gamma-glutamyltransferase increased | R7490 | 1 | van den Elsen, 2015 | **Miscellaneous** |
| Gastrointestinal tract | ? | 1 | Zajicek, 2003 | **Miscellaneous** |
| Headache | R5100 | 9 | Brisbois, 2011<br>Svendsen, 2004<br>Schimrigk, 2017<br>Malik, 2017<br>Ahmed, 2014<br>Esfandyari, 2006<br>Wong, 2012<br>Killestein, 2002<br>van den Elsen, 2015 | **Miscellaneous** |
| Hepatic enzyme increased | R7490 | 1 | van den Elsen, 2015 | **Miscellaneous** |
| Hives/rash | R21H0 | 1 | Brisbois, 2011 | **Miscellaneous** |
| Low blood count | R7990 | 1 | Brisbois, 2011 | **Miscellaneous** |
| Malaise | R5300 | 2 | Ahmed, 2014<br>van den Elsen, 2015 | **Miscellaneous** |
| Miosis | H5700 | 1 | van den Elsen, 2015 | **Miscellaneous** |
| Nausea | R13H0 | 5 | Svendsen, 2004<br>Schimrigk, 2017 Malik,<br>2017 Ahmed, 2014<br>Esfandyari, 2006 | **Miscellaneous** |
| Nausea/Vomiting | R13H0 | 1 | Brisbois, 2011 | **Miscellaneous** |
| Numbness | R2080 | 1 | Esfandyari, 2006 | **Miscellaneous** |
| Other | R6880 | 1 | Killestein, 2002 | **Miscellaneous** |
| Pain | R5200 | 1 | Brisbois, 2011 | **Miscellaneous** |
| Palpitations | R0020 | 1 | Svendsen, 2004 | **Miscellaneous** |
| Presyncope | R5500 | 1 | van den Elsen, 2015 | **Miscellaneous** |
| Restlessness | R4510 | 1 | van den Elsen, 2015 | **Miscellaneous** |
| Sensory loss | R4480 | 1 | van den Elsen, 2015 | **Miscellaneous** |

| | | | | |
|---|---|---|---|---|
| Shortness of breath / fluid on lungs | R0600 | 1 | Brisbois, 2011 | **Miscellaneous** |
| Skin disorder, not otherwise specified | R2380 | 1 | van den Elsen, 2015 | **Miscellaneous** |
| Syncope | R5500 | 1 | van den Elsen, 2015 | **Miscellaneous** |
| Tired / drowsy | R5300 | 1 | Brisbois, 2011 | **Miscellaneous** |
| Unsteady feet | H8190 | 1 | Brisbois, 2011 | **Miscellaneous** |
| Vasovagal | R5500 | 1 | Esfandyari, 2006 | **Miscellaneous** |
| Vertigo | R42H0 | 1 | Schimrigk, 2017 | **Miscellaneous** |
| Weight decrease | R6340 | 1 | Svendsen, 2004 | **Miscellaneous** |
| Back pain | M5480 | 1 | van den Elsen, 2015 | **Musculoskeletal** |
| Increased spasticity | M6290 | 1 | Killestein, 2002 | **Musculoskeletal** |
| Muscle spasms | M6290 | 1 | van den Elsen, 2015 | **Musculoskeletal** |
| Muscle weakness | M6280 | 2 | Svendsen, 2004<br>van den Elsen, 2015 | **Musculoskeletal** |
| Myalgia | M7910 | 1 | Svendsen, 2004 | **Musculoskeletal** |
| Pain in extremity | M7960 | 1 | van den Elsen, 2015 | **Musculoskeletal** |
| Nasopharyngitis | J0000 | 1 | van den Elsen, 2015 | **Respiratory** |
| Pneumonia | J1890 | 2 | Brisbois, 2011<br>van den Elsen, 2015 | **Respiratory** |
| Upper airway infection | J0690 | 1 | Svendsen, 2004 | **Respiratory** |
| Hot flushes | N9580 | 2 | Svendsen, 2004<br>Wong, 2012 | **Urogenital** |
| Renal impairment | N1900 | 1 | van den Elsen, 2015 | **Urogenital** |
| Urge incontinence | N3940 | 1 | van den Elsen, 2015 | **Urogenital** |
| Vaginal discharge | N8980 | 1 | Brisbois, 2011 | **Urogenital** |
